# Short peptides based on the conserved regions of MIEN1 protein exhibit anticancer activity by targeting the MIEN1 signaling pathway

**DOI:** 10.1016/j.jbc.2024.105680

**Published:** 2024-01-23

**Authors:** Amit K. Tripathi, Priyanka P. Desai, Antariksh Tyagi, Jana B. Lampe, Yogesh Srivastava, Michael Donkor, Harlan P. Jones, Sergei V. Dzyuba, Eric Crossley, Noelle S. Williams, Jamboor K. Vishwanatha

**Affiliations:** 1 Department of Microbiology, Immunology and Genetics, University of North Texas Health Science Center, Fort Worth, Texas, USA; 2 Yale Center for Genome Analysis (YCGA), Yale School of Medicine, New Haven, Connecticut, USA; 3 Department of Genetics, University of Texas MD Anderson Cancer Center, Houston, Texas, USA; 4 Department of Chemistry and Biochemistry, Texas Christian University, Fort Worth, Texas, USA; 5 Department of Biochemistry, University of Texas Southwestern Medical Center, Dallas, Texas, USA

**Keywords:** breast cancer, epidermal growth factor (EGF), cancer biology, protein motif, anticancer peptides, epithelial-mesenchymal transition (EMT), pharmacokinetics, peptide design

## Abstract

Migration and invasion enhancer 1 (MIEN1) overexpression characterizes several cancers and facilitates cancer cell migration and invasion. Leveraging conserved immunoreceptor tyrosine-based activation motif and prenylation motifs within MIEN1, we identified potent anticancer peptides. Among them, bioactive peptides LA3IK and RP-7 induced pronounced transcriptomic and protein expression changes at sub-IC50 concentrations. The peptides effectively inhibited genes and proteins driving cancer cell migration, invasion, and epithelial-mesenchymal transition pathways, concurrently suppressing epidermal growth factor-induced nuclear factor kappa B nuclear translocation in metastatic breast cancer cells. Specifically, peptides targeted the same signal transduction pathway initiated by MIEN1. Molecular docking and CD spectra indicated the formation of MIEN1-peptide complexes. The third-positioned isoleucine in LA3IK and CVIL motif in RP-7 were crucial for inhibiting breast cancer cell migration. This is evident from the limited migration inhibition observed when MDA-MB-231 cells were treated with scrambled peptides LA3IK SCR and RP-7 SCR. Additionally, LA3IK and RP-7 effectively suppressed tumor growth in an orthotopic breast cancer model. Notably, mice tolerated high intraperitoneal (ip) peptide doses of 90 mg/Kg well, surpassing significantly lower doses of 5 mg/Kg intravenously (iv) and 30 mg/Kg intraperitoneally (ip) used in both *in vivo* pharmacokinetic studies and orthotopic mouse model assays. D-isomers of LA3IK and RP-7 showed enhanced anticancer activity compared to their L-isomers. D-LA3IK remained stable in mouse plasma for 24 h with 75% remaining, exhibiting superior pharmacokinetic properties over D/L-RP-7. In summary, our findings mark the first report of short peptides based on MIEN1 protein sequence capable of inhibiting cancer signaling pathways, effectively impeding cancer progression both *in vitro* and *in vivo*.

Discovering novel strategies for cancer treatment is a pressing priority within the realm of cancer therapeutics. While chemotherapy remains the prevailing approach, its efficacy is often hampered by its indiscriminate toxicity toward normal cells and the necessity for high drug dosages. This frequently leads to the emergence of chemoresistance, a major factor in treatment failures of both breast and prostate cancer cells (1, 2). Moreover, the highly heterogeneous nature of triple-negative breast cancer (TNBC) and the absence of three key receptors present a significant hindrance to successful treatment (3). Anticancer peptides (ACPs) have been shown to selectively inhibit tumor cell migration and invasion, or suppress angiogenesis and thus are less likely to cause chemoresistance (4, 5). Peptides derived from naturally occurring proteins are known to counteract the signaling pathways of their parental proteins, demonstrating potent biological activities (6, 7, 8, 9).

Pioneering research by Dasgupta *et al*. initially showed the significance of migration and invasion enhancer 1 (MIEN1), formerly known as C17orf37, in promoting cancer cell migration and invasion *via* nuclear factor kappa B (NF-κB) pathway activation (10). Subsequent investigations by Chang *et al*. extended these findings in human prostate carcinoma cells (11). MIEN1, a compact protein of 115 amino acids with a molecular weight of 12 kDa, was initially detected in breast and colon cancer cells (12). Its expression diverges dramatically between normal and cancerous cells, with heightened levels correlating positively with advanced breast cancer stages (12, 13). This distinct expression pattern positions MIEN1 as a potential biomarker and therapeutic target for tumors (14). MIEN1 contains two important functional motifs: the immunoreceptor tyrosine-based activation motif (ITAM) is a catalyst for downstream signaling pathways (15), and the prenylation motif imparts migratory and invasive capacity to the protein and promotes protein-mediated metastasis (13). We have identified short peptides derived from the ITAM and prenylation motifs of MIEN1 as potential ACPs for cancer therapy.

## Results

### Peptide design

**Figure 1:**
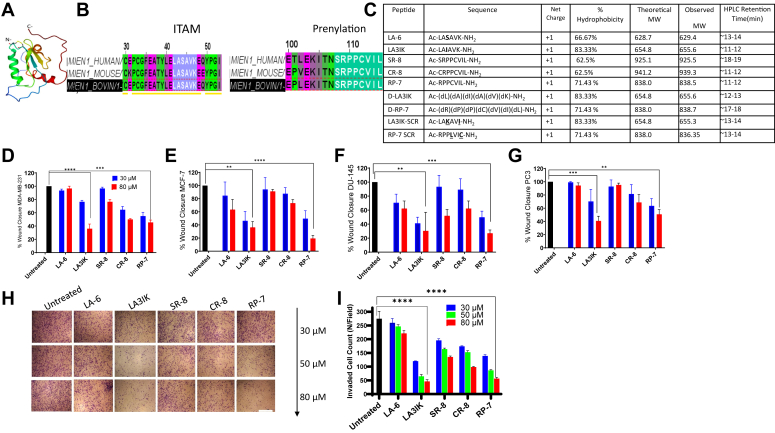
**MIEN1 has a conserved functional immunoreceptor tyrosine-based activation motif and prenylation motif.***A*, solution structure of MIEN1 protein. *B*, schematic representation of the ITAM and CAAX-prenylation motif on MIEN1 protein in three different species. *C*, peptide sequences and their analogs along with biophysical parameters. Amino acid residues that were substituted/interchanged were highlighted in *bold* and *underlined*. *D*, wound healing assay with MDA-MB-231 (*E*) MCF-7, (*F*) DU-145, and (*G*) PC-3 cells. Microscopic visualizations supporting the graphs are provided in Figure S4. *H*, dose-dependent inhibition of MDA-MB-231 cell invasion through the transwell after peptide treatment. Representative images for the untreated and peptide-treated cells at 10× magnification. The scale bar represents 1000 μm. *I*, graph represents the number of invaded cells mean ± SD from random fields at each peptide concentration. Wound healing assays were performed at least twice at two different concentrations while the transwell invasion assay was performed in triplicates. Data were analyzed by one-way ANOVA ∗∗*p* <0.01, ∗∗∗*p* < 0.001 and ∗∗∗∗*p* < 0.0001. ITAM, immunoreceptor tyrosine-based activation motif; MIEN1, migration and invasion enhancer 1.

The Protein Data Bank structure of MIEN1 has a canonical ITAM motif of 18-amino acids (YxxI(6–8)YxxL) which starts with a tyrosine residue and is separated from a leucine or isoleucine residue by any two other amino acids in between, giving the YxxL/I sequence (Fig. 1, *A* and *B*) (15). We chose a hexamer LASAVK from this conserved region to serve as a template for peptide design. Studies have shown that ACPs that contain two main biophysical parameters, high hydrophobicity and cationicity, can selectively target cancer cells by electrostatically interacting with the anionic lipids present in the plasma membrane of cancer cells (16, 17, 18). To achieve this, the third-positioned serine in LA-6 was replaced with hydrophobic amino acid isoleucine to design the first analog LAIAVK. MIEN1 also contains a functional prenylation (CAAX) motif in form of CVIL which is posttranslationally modified by protein geranylgeranyltransferase-I (13). Geranylgeranylation of MIEN1 at the CVIL motif enables the protein to associate with the inner leaflet of the plasma membrane and induces filopodia formation, promoting migration of cancer cells (15). Three more peptides were designed from the prenylation motif of the MIEN1 protein. A cysteine analog was also designed in which serine was replaced with cysteine. The peptides were named as per the first two amino acid residues of each peptide (Fig. 1*C*). The numeral indicates the length of the peptide. The peptides were purified to ∼95% homogeneity. Following the establishment of the biological activity of LA3IK and RP-7, we designed D-isomers of both peptides with the objective of enhancing their anticancer effectiveness, while concurrently bolstering their stability. The inclusion of D-amino acids, characterized by their distinct stereochemistry causes improved anticancer properties and better stability in the living system. To affirm the positional importance of LA3IK and RP-7, two scrambled peptides were synthesized: one involving the interchange of the third isoleucine and terminal lysine in LA3IK, and the other disturbing the -CVIL motif of RP-7 by interchanging the positions of cysteine and leucine. These modifications and controls were implemented to investigate the specific contributions of individual amino acids and motifs to the peptides' biological activities. The HPLC profiles and the molecular masses of purified peptides were determined LC/MS Agilent 6125B Single Quadrupole LC/MS (Fig. S1).

### MIEN1-derived peptides and their analogs displayed favorable toxicological parameters

The calculated IC50 values for normal NIH-3T3 cells were notably higher compared to MDA-MB-231, MCF-7, PC-3, and DU-145 cells (Fig. S2 & Table S3), underscoring the intrinsic cancer cell-selectivity exhibited by LA3IK and RP-7 peptides. It is worth emphasizing that as all experimental conditions were maintained at sub-IC50 concentrations, the observed inhibition attributed to the peptides is associated with their ability to hinder crucial signaling pathways implicated in cancer advancement, rather than exerting generalized cytotoxic effects on the cells. To further ascertain the safety and potential of bioactive peptides LA3IK and RP-7, an *in silico* toxicological assessment was conducted using Pro-Tox-II, a virtual platform renowned for predicting the toxicities of small molecules (19). The predicted LD_50_ values for both peptides stood at 3000 mg/kg, thereby positioning these compounds within a favorable and relatively safe range spectrum of drug design and development. Notably, the predictive algorithm also indicated the peptides' inactivity across diverse toxicity endpoints. This encompassed acute toxicity, hepatotoxicity, cytotoxicity, carcinogenicity, mutagenicity, and immunotoxicity, as well as their impact on adverse outcomes within Tox21 pathways and toxicity targets. (Fig. S3).

### RP-7 and LA3IK suppress the migratory and invasive capacity of MDA-MB-231 cancer cells

Sub-IC50 concentrations of RP-7 and LA3IK markedly curtailed the wound closure rate of highly migratory MDA-MB-231 cells. Relative to untreated cells, the percent wound closure for LA3IK stood at 76% and 36% for lower and higher concentrations respectively, while for RP-7, the wound closures were 55% and 45% for lower and higher peptide doses (Fig. 1*D*). Both peptides retained their activity in other cell types like MCF-7, DU-145, and PC-3 cells (Fig. 1, *E*, *F,* and *G* (Fig. S4).

The inhibitory influence of these peptides was further discernible in their impact on cellular invasiveness. In addition to migration, cancer cells invade healthy tissue in the host by degrading extracellular matrix proteins and transversing extracellular membranes by the process of invasion. LA3IK and RP-7 resulted in a dose-dependent reduction in the number of invaded MDA-MB-231 cells (Fig. 1, *H* and *I*). The other peptides of the series LA-6, SR-8, and its cysteine analog CR-8 exhibited limited inhibition of migration and invasion at equimolar peptide concentrations. Interestingly, the D-isomers of LA3IK and RP-7 displayed improved efficacy in blocking MDA-MB-231 cell migration. Specifically, when treated with identical concentrations, the D-LA3IK demonstrated a 30.2% decrease in cell migration, while D-RP-7 displayed an 18.9% reduction in cellular migration as compared to their L counterparts, as detected through the wound healing assay. LA3IK SCR and RP-7 SCR showed nonsignificant inhibition of migration compared to untreated MDA-MB-231 cells. Besides this, both LA3IK SCR and RP-7 SCR exhibited reduced antimigratory activity across three distinct cancer cell lines (MCF-7, DU-145, and PC-3) compared to their counterparts LA3IK and RP-7 in a wound healing assay conducted at equivalent peptide concentrations. This observation underscores the significance of the amino acid sequence in both bioactive peptides, indicating their pivotal role in impeding the migration of cancer cells. (Fig. S5, *E*–*H*).

### RNA-seq data reveals pronounced downregulation of cancer progression-related genes and upregulation of inhibitory genes

**Figure 2:**
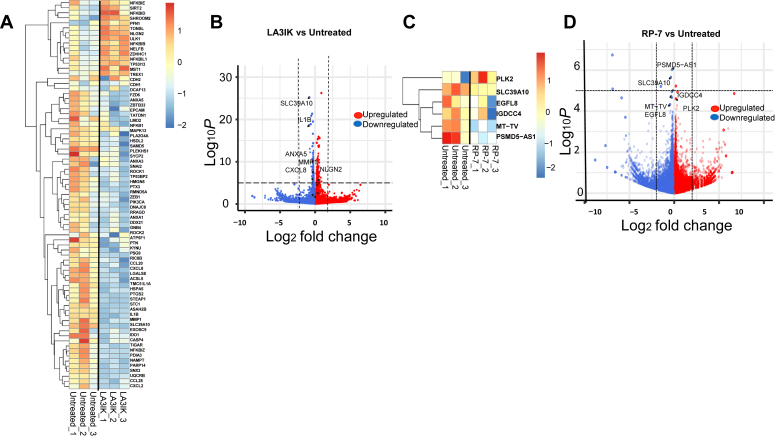
**LA3IK and RP-7 suppressed cancer-promoting genes, activated tumor-suppressing genes, and reversed EMT markers in MDA-MB-231 TNBC cells.** Three biologically independent RNA samples per group were sequenced. DESeq2 was used to calculate the fold-change and *p*-value). Upregulated and downregulated genes were identified by the cutoff of *p* < 0.05. z-score of log2 TPM values were plotted in a heatmap. Volcano plot shows the distribution of differentially expressed genes. *A* and *B*, show the heatmap and volcano plot of selected genes after 48 h exposure to LA3IK. *C* and *D*, show the heatmap and volcano plot of selected genes after 48 h exposure to RP-7. Peptide concentration: 90 μM. EMT, epithelial-mesenchymal transition; TNBC, triple-negative breast cancer; TPM, transcript per million.

The RNA-seq analysis was performed on peptide-treated MDA-MB-231 cells to identify the differentially expressed genes (DEGs). In LA3IK-treated samples, a significant downregulation was observed among genes central in cancer-related inflammation and epithelial-mesenchymal transition (EMT), exemplified by the reduced expression of IL1B, CXCL-8, matrix metalloproteinase 1 (MMP-1), and Zeb-1 (Fig. 2*A*). Conversely, an upregulation emerged in genes associated with the inhibition of breast cancer progression, including SIRT2, MST1, and ULK1, indicating the therapeutic potential of the peptide (Fig. 2, *A* and *B*) (20, 21). Genes belonging to the proinflammatory gene signature, as well as some that are involved in EMT for MDA-MB-231 cells were examined. In RP-7 treated cells, an enrichment of tumor suppressor genes namely, PLK2, and a decrease in prognostic markers like epidermal growth factor-like protein 8 were observed (22, 23) (Fig. 2, *C* and *D*).

### LA3IK and RP-7 peptides exert their inhibitory activity on DU-145 prostate cancer cells

**Figure 3:**
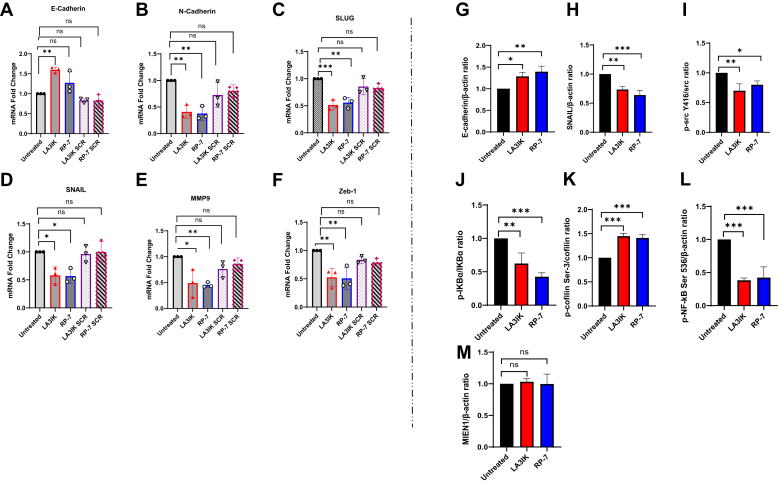
**Quantitative real-time PCR analysis and Western Blots of different genes in DU-145 after peptide treatment.***A*–*F*, changes in the mRNA expression levels of EMT-related genes were evaluated using real-time RT-PCR after 48 h treatment with bioactive peptides LA3IK and RP-7 and their scrambled analogs LA3IK SCR and RP-7 SCR as controls. The expression of epithelial marker E-cadherin was upregulated in LA3IK and RP-7 treated cells and downregulated for N-cadherin, SLUG, SNAIL, MMP-9, and Zeb-1. 18S RNA transcript levels were used for internal control. *G*–*M*, whole cell lysates are made 48 h posttreatment with LA3IK and RP-7. E-cadherin and SNAIL are increased and decreased, respectively, when probed with antibodies specific for these proteins as compared to the untreated. Phosphorylation of NF-κB and IκBα at the key serine residues was also inhibited in peptide-treated DU-145 cells in comparison to the untreated cells. Densitometry was determined and normalized to total protein or β-actin. Peptide concentration: 90 μM. All the assays were performed in triplicates and the results are expressed as means ± SD. Data were analyzed by one-way ANOVA. ∗*p*< 0.05; ∗∗*p* < 0.01; ∗∗∗*p* <0.001; ns = Not significant. Full-length representative blots were presented in Figure S7(1). EMT, epithelial-mesenchymal transition; MMP-9, matrix metalloproteinase 9; NF-κB, nuclear factor kappa B.

The efficacy of LA3IK and RP-7 as potential anticancer agents was demonstrated in DU-145 prostate cancer cells. Application of these peptides led to a disruption in the expression of genes and proteins associated with crucial processes such as migration, EMT and NF-κB activity (illustrated in Fig. 3). Notably, we observed a significant increase in E-cadherin expression accompanied by a corresponding decrease in N-cadherin and SNAIL expression, both at the gene and protein levels. Other transcription factors such as SLUG, SNAIL, Zeb-1, and MMP-9, which regulate EMT processes, were also downregulated at the mRNA levels. The suppression of N-cadherin and transcription factors such as SLUG and SNAIL, coupled with the upregulation of E-cadherin, is a common feature of the inhibition of EMT induction. Except for SNAIL, LA3IK SCR and RP-7 SCR demonstrated statistically nonsignificant inhibition of the majority of the genes examined in the PCR analysis. However, LA3IK SCR-treated DU-145 cells exhibited statistically diminished gene expression in Zeb-1 and SNAIL, whereas RP-7 SCR displayed reduced expression of SLUG and Zeb-1. (Fig. 3, *A*–*F*). Moreover, a reduction in src phosphorylation, coupled with an augmentation in cofilin phosphorylation, was evident in Western blot assays, signifying a concurrent reduction in the migratory potential of the DU-145 cells. Intriguingly, the exposure of DU-145 cells to LA3IK and RP-7 resulted in a pronounced decrease in the phosphorylation of NF-κB p65 at serine-536. This event led to a subsequent decline in NF-κB transcriptional activity through the sequestration of cytoplasmic NF-κB inhibitor alpha (IκBα) complexes. The attenuation of serine-32 phosphorylation in IκBα further underscored the modulation of NF-κB signaling by the peptides. The MIEN1 protein levels were unaffected by the peptide treatment (depicted in Fig. 3, *G*–*M*).

### LA3IK and RP-7 inhibit proteins involved in MDA-MB-231 and DU-145 cancer cell migration and actin dynamics

**Figure 4:**
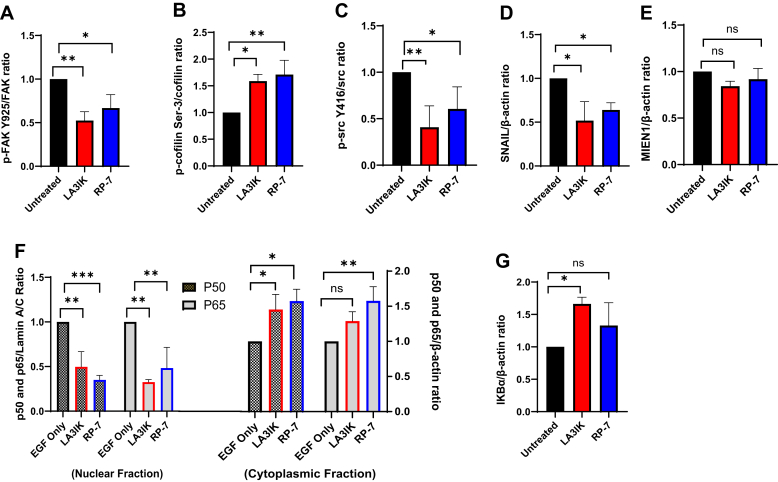
**LA3IK and RP-7 inhibit the proteins involved in MIEN1 signaling pathway in MDA-MB-231 cells.***A*–*D*, densitometry analysis of LA3IK and RP-7 treated MDA-MB-231 cells. Cells were exposed to the peptides and subjected to Western blot analysis with the indicated antibodies. *E* and *F*, peptides also inhibit the EGF-mediated NF-κB nuclear translocation in MDA-MB-231. Cytoplasmic and nuclear cell lysate extracts of EGF only and EGF plus peptide-treated cells were probed for p65 and p50. Lamin A/C and β-actin were used as nuclear and cytoplasmic loading control respectively. Densitometry was determined with immunoblots (N = 3) and normalized to total protein or β-actin for whole cell lysates and cytoplasmic fraction of peptide-treated MDA-MB-231 cells. In case of nuclear lysates, the immunoblots were normalized with Lamin A/C. Bar graphs are densitometric data (means ± SD). Data were analyzed by one-way ANOVA. ∗*p* < 0.05; ∗∗*p* <0.01; ∗∗∗*p* <0.001; ns = Not significant. EGF concentration: (10 ng/ml); Peptide concentration: 90 μM. Full-length representative blots are presented in Figure S7(2) and S8(1). EGF, epidermal growth factor; MIEN1, migration and invasion enhancer 1; NF-κB, nuclear factor kappa B.

The higher level of MIEN1 enhances tumor growth and actin reorganization inducing phosphorylation of focal adhesion kinase (FAK) at tyrosine-925 and reducing cofilin phosphorylation at serine-3. These two changes increase the migration of breast cancer cells (24). To elucidate mechanisms of peptide-induced inhibition of MIEN1-regulated signaling pathways, we examined cell adhesion and actin dynamics pathways following the peptide exposure in MDA-MB-231. While the MIEN1 proteins remained unaltered in response to the peptide treatments, an intriguing observation emerged: the MIEN1 downstream signaling pathway, which involves the phosphorylation of FAK at tyrosine- 925, exhibited inhibition in MDA-MB-231 cell lysates treated with LA3IK and RP-7. This inhibition signifies the suppression of a key protein within the MIEN1 signaling cascade. The actin dynamics regulated by MIEN1 to facilitate migration were also affected by LA3IK and RP-7 as the accumulation of phosphorylated cofilin (inactive cofilin) was observed in peptide-treated whole-cell lysates of both MDA-MB-231 and DU-145 cells. The phosphorylation of src at tyrosine-416 was also inhibited by LA3IK and RP-7 which downregulated the kinase activity of src (Fig. 4, *A*–*D*). The D-isomers of LA3IK and RP-7 demonstrated enhanced potency than their corresponding L-peptide counterparts. Specifically, the D-isomer of LA3IK exhibited 34.2% and 48.8% greater inhibition of FAK and SRC phosphorylation, respectively. In case of RP-7, there was a 24.2% and 29.5% increase in the inhibition of p-FAK and p-SCR, as indicated by the mean values of immunoblot densitometry (Fig. S5, *B*–*D*).

### LA3IK and RP-7 reduced the EGF-mediated NF-κB nuclear translocation in MDA-MB-231 cells

The transcription factor NF-κB is constitutively active in breast cancer, and its transcriptional activity is enhanced before malignant transformation in the breast (25). It has also been postulated to be a vital marker for EMT and invasiveness in breast cancers with its expression being induced by the MIEN1 gene (11, 26, 27). We evaluated the possible effects of MIEN1-derived peptide and its analogs on the nuclear translocation of NF-κB. The inhibitory ability was examined in the presence of epidermal growth factor (EGF), a well-known NF-κB activator in this TNBC cell line (28). The densitometric analysis of the immunoblots suggested that the nuclear lysates of LA3IK and RP-7 had lesser translocation of p50 and p65 than the EGF-alone treated MDA-MB-231 cells (Fig. 4*E*). Additional evidence supporting the inhibition of NF-κB nuclear translocation was obtained through densitometric analysis of cytoplasmic IκBα. The results revealed higher levels of IκBα in the cytoplasmic fractions of cells treated with peptides than those treated with EGF alone in MDA-MB-231 cells (Fig. 4*F*).

### LA3IK and RP-7 displayed observable indications of their interaction with MIEN1

Despite having a short amino acid sequence, LA3IK attained secondary structure conformation in the MIEN1 protein microenvironment. CD experiments with LA3IK showed an apparent mix of β-type structures, that is, turns and strands as judged by the difference spectra. On the other hand, RP-7 despite having two prolines that are known helix breakers exhibited a nonlinearity of the difference spectra and displayed appreciable deviation from the zero-line (Fig. S6*A*). All the other peptides remained randomly coiled both in the aqueous and MIEN1 protein microenvironment. For the bioinformatics studies, binding pocket predictions were done by utilizing CASTp server based on surface area and volume. Docking simulations suggest potential interactions between MIEN1 and both LA3IK and RP-7. The RP-7 peptide demonstrates stronger binding within pocket1, while LA3IK predicts binding within pocket2 of the MIEN1 protein (Fig. S6, *B*–*E*). RP-7 docking indicates a disulfide bond formation between cysteine-4 of RP-7 peptide and cysteine-114 of MIEN1. In contrast, LA3IK lacks such bonding. RP-7-MIEN1 interaction involves 14 hydrogen bonds and a disulfide bond, while LA3IK forms four hydrogen bonds and eight closely positioned atoms for hydrophobic interactions (Fig. S6, *F*–*G*). DeltaG values are −15 Kcal/mol for RP-7 and −13 Kcal/mol for LA3IK, revealing distinct interaction residues. RP-7 binding to MIEN1 is primarily mediated by the C terminus, while LA3IK binding involves both termini (Fig. S6, *F* and *G*).

### Peptide stability and pharmacokinetic studies

**Figure 5:**
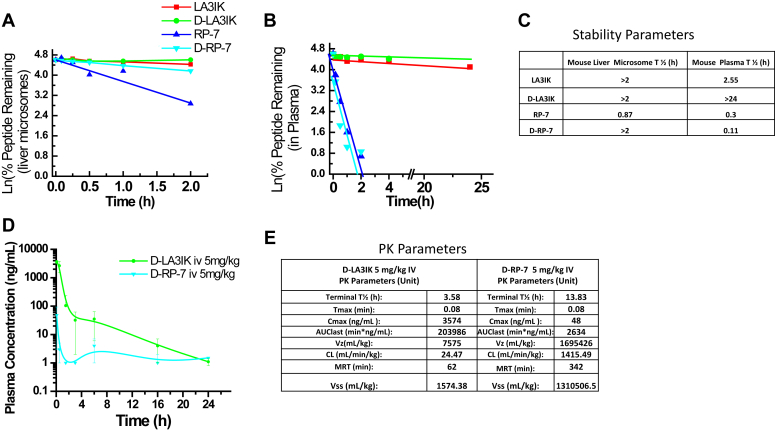
**Stability assay and PK studies of (D/L) isomers of LA3IK and RP-7 in CD-1 mice.***A*, graph shows peptide disappearance with time in the presence of (*A*) mouse liver microsomes. *B*, mouse plasma. *C*, table showing half-life (T ½) calculated as T ½ = 0.693/slope. *D*, mean plasma concentrations over time profile for D-LA3IK and D-RP-7 after 5 mg/kg iv. *E*, tables under the graphs are the estimated PK parameters. PK, pharmacokinetics.

Prior to initiating *in vivo* studies involving LA3IK and RP-7, we assessed compound stability through incubation with mouse liver microsomes and mouse plasma. The half-life (T1/2) of LA3IK in microsomes demonstrated robust stability, exceeding 2 h, while that of RP-7 was relatively shorter, measuring 0.87 h (52.3 min) (Fig. 5*A*). Notably, RP-7 exhibited expected instability in mouse plasma. The design of D-isomers for LA3IK and RP-7 proved strategically advantageous, particularly evident in the case of LA3IK. This approach notably improved its plasma stability, with a remarkable transformation from an initial T_1/2_ of 2.55 h for the L-isomer of LA3IK to an impressive >24 h for its D-isomer counterpart as calculated by Ln (%remaining) graph (Fig. 5, *B* and *C*). The stability was also extended to the liver microsomal stability assay, where the D-isomer of LA3IK demonstrated significant and promising improvements in microsomal stability. After a 2-h incubation period, a full 100% of the D-LA3IK remained intact, a contrast to the 83.7% observed for L-LA3IK. Likewise, in the case of D-RP-7, a substantial 64.4% of the intact peptide was detected within mouse microsomes, presenting a stark contrast to the mere 17.7% detection exhibited by L-RP-7 under similar conditions (Fig. 5*A*). To corroborate these findings, *in vivo* pharmacokinetic analyses were conducted, revealing a substantial 80-fold increase in total exposure for D-LA3IK compared to D-RP-7 Figure 5*E*). However, for the subsequent *in vivo* studies, the selection of L isoforms for both LA3IK and RP-7 was purposeful. This choice was driven by their enhanced biological relevance and the consideration that they are less likely to induce immunogenicity or interfere with normal metabolic pathways.

### Administration of LA3IK and RP-7 reduced tumor burden in an orthotopic model of breast cancer

**Figure 6:**
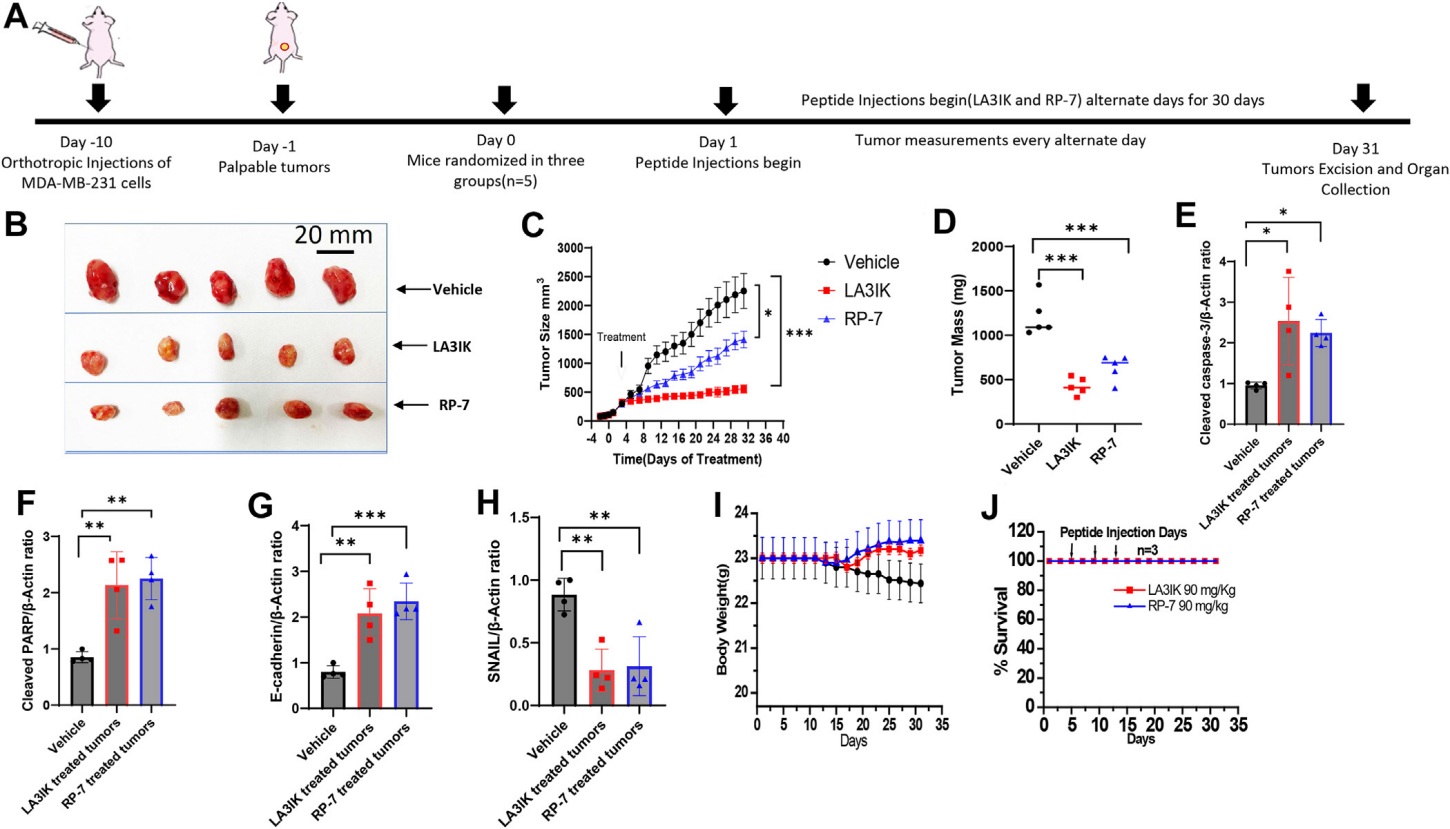
***In vivo* efficacy of LA3IK and RP-7 in nude mice bearing MDA-MB-231 orthotopic tumors.***A*, timeline of the *in vivo* experiment. MDA-MB-231 cells were implanted orthotopically into the second thoracic mammary fat pad of nude mice (n = 5). After 10 days, tumor-bearing mice were grouped and treated with LA3IK and RP-7 by intraperitoneal injections at a dose of 30 mg/kg every alternate day. The control mice received vehicle only. *B*, at the end of the experiments, representative tumors were removed and photographed. *C*, the tumor growth did not decrease in the control group, whereas the LA3IK and RP-7 decreased tumor growth significantly. The tumor sizes were measured by a digital caliper and shown as mean volume  ±  SDs every alternate day for 30 days. Unpaired t-test, LA3IK (30 mg/Kg), ∗∗∗*p* < 0.001 and RP-7 (30 mg/Kg), ∗*p* < 0.05. The statistical analysis was performed on GraphPad Prism 9.4.0. *D*, individual tumor weight of each mouse in the LA3IK and RP-7 treated group in comparison with the control group after 30 days of treatment N = 5). *E*–*H*, Western blots of cell lysates prepared from the tumors of untreated tumor (Vehicle) *versus* LA3IK and RP-7 for apoptosis-associated proteins. Number of tumors taken for study = 4 per group. Densitometry was determined and normalized to β-actin. Bar graphs are densitometric data (means ± SD). Data analyzed by one-way ANOVA. ∗*p* < 0.05,∗∗*p* <0.01 and ∗∗∗*p* < 0.001. Full-length representative blots are in Figure S8(2). *I*, body weight loss was observed in the control group after 20th day of the experiment. *J*, *in vivo* toxicity of LA3IK and RP-7: athymic nude mice were injected intraperitoneally with three times the experimental peptide dose (90 mg/Kg of body weight) on day 5, day 10, and day 15. The mice were monitored for changes in body weights as a surrogate marker for toxicity. All mice (N = 3) were alive after 30 days. No body weight loss or other signs of distress were observed.

LA3IK and RP-7 were injected in a group of mice bearing tumors grown from MDA-MB-231 cells. In this model, MDA-MB-231 tumor cells were inoculated into the mammary fat pads of female mice, and when the tumors became palpable, treatment started with LA3IK and RP-7 at 30 mg/Kg on alternate days. Four weeks post-inoculation, the control group showed a mean tumor size of 2254.32 mm^3^ *versus* 554.73 mm^3^ in LA3IK-treated mice (*p* < 0.001). Thus, treatment with LA3IK resulted in reduced tumor volume by 75.3%. Similarly, in the RP-7 treated mice, the mean tumor size on termination day was 1410.51 mm^3^ in comparison to 2254.32 mm^3^ in the untreated (*p* < 0.05), which shows a reduction in tumor volume of 37.4%. Representative photographs of the tumors post-excision are illustrated in Figure 6*B*. The results showed that both LA3IK and RP-7 were quite effective in suppressing tumor growth in mice in the *in vivo* system which is an indicator of their therapeutic potential (Fig. 6, *C* and *D*).

The peptides administered to mouse tumors resulted in a noticeable increase in the levels of cleaved caspase-3 and cleaved poly(ADP-ribose) polymerase (PARP), as observed in the western blot assays when compared to the control groups (Fig. 6, *E* and *F*). Cleaved caspase-3 plays a pivotal role in transmitting apoptotic signals through its enzymatic actions on downstream targets, including PARP and other substrates. PARP, which is an ADP-ribosylating enzyme, is essential for initiating various types of DNA repair processes (29). However, when it is cleaved by activated caspase-3, PARP loses its functionality, leading to the suppression of DNA repair mechanisms. This, in turn, may promote cell death through both caspase-dependent and caspase-independent pathways of apoptosis (30). Moreover, immunoblots of treated tumors revealed an elevation in E-cadherin levels and a concurrent reduction in SNAIL expression(Fig. 6, *G*–*H*).To check the potential toxicity of peptides alone, two groups of mice received three intraperitoneal injection (i.p.) doses of 90 mg/Kg of LA3IK and RP-7 which is markedly higher than the 5 mg/Kg i.v. and 30 mg/Kg i.p. doses used in *in vivo* pharmacokinetics and orthotopic mouse model assays, respectively The mice did not display any signs of toxicity and were alive on the termination day of the experiment (Fig. 6, *I* and *J*).

## Discussion

This study explores harnessing conserved ITAM and prenylation motifs of MIEN1 to identify peptides inhibiting its signaling pathway for potent anticancer effects, contrasting with miRNA-targeting approaches used previously (31, 32, 33). Single amino acid substitutions have been made in naturally occurring ACPs to elucidate their pivotal roles in biological activity (34, 35). LA-6, a native ITAM motif-derived peptide, transformed into LA3IK through a serine-to-isoleucine substitution, enhancing hydrophobicity by 16.6%. This hydrophobicity enhancement stands as a key contributor to the peptides' anticancer activity (16, 17). Similarly, deletion of N terminus serine from SR-8 led to the design of potent RP-7 peptide, surpassing SR-8 in terms of targeting the MIEN1 signaling pathway. Both peptides showed selective anticancer attributes with high safety margins in toxicology assessments. Cancer cell migration facilitates the movement of primary tumors into surrounding tissues, while the invasion process enables cancer cells to penetrate nearby tissues, often crossing tissue boundaries and entering blood vessels or lymphatic vessels. Both LA3IK and RP-7 demonstrated inhibitory effects on cancer cell migration, invasion, and genes related to cancer progression and EMT in DU-145 and MDA-MB-231 cells. Besides, the mRNA levels of other EMT regulators such as SLUG, SNAIL, and Twist that are directly linked to aggressive tumor characteristics and unfavorable outcomes in individuals with prostate cancer were also downregulated (36). RNA-seq analysis of LA3IK-treated MDA-MB-231 cells revealed interconnections between EMT, cell proliferation, and inflammation pathways. Notably, both peptides downregulated proinflammatory genes and inhibited EMT processes, suggesting broader implications for various cancer types (37, 38). RP-7 downregulated prognostic markers (epidermal growth factor-like protein 8) and upregulated tumor suppressor genes (PLK2) in breast cancer cells, along with suppressing SLC39A10 involved in cancer progression. The inhibition of EMT genes was more pronounced in DU-145 cells, where both LA3IK and RP-7 reduced N-cadherin and EMT transcription factors (SLUG, SNAIL, MMP-9, and Zeb-1), and increased E-cadherin levels. LA3IK and RP-7 treatments in MDA-MB-231 cells hindered FAK phosphorylation at tyrosine-925, reducing src phosphorylation at tyrosine-416 and increasing cofilin's serine-3 phosphorylation. This dual modulation inhibited src-driven proliferation, migration, and disrupted actin dynamics, impeding overall tumor dissemination (39, 40, 41). Nonphosphorylated cofilin is an activated form of cofilin and plays a crucial role in promoting actin polymerization and directional cell motility (42). The peptides also diminished NF-κB activity in DU-145 cells and reduced EGF-induced NF-κB nuclear translocation in MDA-MB-231 cells. This holds therapeutic significance for TNBC, targeting EGF receptor-mediated signaling, an area where current treatments like cetuximab have limited success (43). The peptides' effect on the inhibition of NF-κB signaling pathways is broad and multifaceted (44, 45). On the one hand, it cripples the cancer cell migration and invasion and on the other hand, it inhibits the NF-κB induced EMT in cancer cells (26).LA3IK and RP-7 exhibited distinct interactions with the MIEN1 protein, evident in noticeable deviations in CD spectra. LA3IK attained β-sheet and turns conformation in the presence of MIEN1 while RP-7 showed weaker deviations, possibly influenced by two proline residues (46, 47, 48). Docking simulations for RP-7 indicate a strong, complex binding with MIEN1 involving 14 hydrogen bonds and a disulfide bond. The participation of MEIN1 residue cysteine-114 suggests that RP-7 targets the prenylation motif of MIEN1. In contrast, LA3IK's interaction implies a broader binding interface, potentially impacting the protein's overall conformation. The D-isomers and scrambled analogs, namely LA3IK SCR and RP-7 SCR, served two key purposes: D-isomers displayed improved metabolic stability and anticancer activity, while scrambled analogs with swapped amino acid positions highlighted the significance of the third leucine and the prenylation motif CVIL in their anticancer activity. For the bioanalysis, LC/MS methods were preferred over immunological approaches due to time and cost considerations (49). Pharmacokinetic assays corroborated the improved stability and enhanced pharmacokinetic parameters of D-LA3IK and D-RP-7. These two attributes enhance the drugs’ bioavailability and ensure prolonged circulation in the body, allowing for sustained therapeutic effects and increased chances of reaching the target tumor sites.

In an orthotopic mouse model of breast cancer, LA3IK and RP-7 led to a substantial 75.39% reduction in tumor volume within the LA3IK-treated mice group, meeting partial response criteria (50, 51). The RP-7-treated mice group exhibited a 37.43% decrease in tumor volume. This decline in tumor volumes was concomitant with elevated levels of apoptosis-related proteins in the peptide-treated groups. Notably, probing the proteins derived from these tumors with specific antibodies revealed substantial amounts of cleaved caspase-3, cleaved PARP, and E-cadherin suggesting some degree of involvement of apoptosis due to the peptide treatments. Alongside this, significant decreases in EMT marker SNAIL were also noted. These changes collectively contributed to the halting of tumor growth. Both peptides exhibited excellent tolerability at a high intraperitoneal dose of 90 mg/kg in athymic mice, showing no significant toxicity. In conclusion, the bioactive peptides LA3IK and RP-7, based on MIEN1 protein sequence effectively impeded migration and invasion and displayed anticancer activity in the living system.

## Experimental procedures

### Cell lines and peptide treatments

MDA-MB-231, MCF-7, DU-145, PC-3, and NIH-3T3 cell lines were procured from the American Type Culture Collection and used within ten consecutive passages to ensure consistent cell behavior. MDA-MB-231, MCF-7, and NIH-3T3 cells were maintained at 37 °C and 5% CO_2_ in Dulbecco's modified Eagle's medium (DMEM) high glucose (Hyclone) supplemented with 10% fetal bovine serum (FBS) and 1% Antibiotic-Antimycotic (Gibco), while DU-145 and PC-3 cells were grown in RPMI-1640 supplemented with the same concentration of FBS and Antibiotic-Antimycotic. The media was replenished every 2 days. Cells were checked for *mycoplasma* at regular intervals using a PCR-based *Mycoplasma* detection kit plasmocin prophylactic (InvivoGen). In all experiments, cells were maintained in serum-free DMEM for 6 h before peptide treatment. Peptide treatment was done in the media containing 0.1% fetal bovine serum for 48 h in all the experiments unless otherwise stated.

### Peptide synthesis

Peptides were synthesized using Fmoc (N-(9-fluorenyl) methoxycarbonyl)/tBu solid-phase peptide synthesis chemistry, with Fmoc-linker AM resin serving as the supportive matrix. For purification, a reversed-phase preparative HPLC technique was used, utilizing a C18 column (250 × 4.6 mm) and a suitable gradient of water and acetonitrile, in the presence of 0.1% TFA. Molecular mass was confirmed through MALDI-TOF analysis. The amino acid sequences of all peptides used in this study are presented in Figure 1*C* and Table S1.

### Antibodies, primers, and protein

The p-FAK Tyr925 (cat #3284), FAK (cat #3285), p-src Tyr416 (cat #59548), src (cat #2109), p-cofilin Ser 3 (cat #3311), cofilin (cat #5175), NF-κB1 p105/p50 (cat #3035), NF-κB p65 (cat #8242), SNAIL (cat #3879), cleaved PARP (cat #5625), cleaved Caspase-3 (cat #9664), IκBα (cat #4814), Lamin A/C (cat #2032), p-NF-κB p65-Ser536 (cat #3033), and p-IκBα-Ser32 (cat #2859) antibodies were procured from Cell Signaling Technology and were used at 1:1000 dilution; β-actin (cat #sc-47778) antibody was from Santa Cruz Biotechnology and was used at 1:2000 dilution. E-cadherin (cat #610181) was from BD Biosciences and was used at 1:1000 dilution. MIEN1 antibody (cat #H00084299-M02) was from Abnova and was used at 1:2000 dilution. Human recombinant protein EGF was from Gibco (cat #PHG0311). MIEN1 protein was custom produced by Biomatik. The PCR primers were synthesized by Integrated DNA Technologies. The primer sequences are given in Table S2.

### Cell viability assay/IC50 determination and virtual toxicology assessment

Cell viability was determined by 3-[4,5-dimethylthiazol-2-yl]-2,5 diphenyl tetrazolium bromide (MTT) assay as described earlier (52). ProTox-II "Prediction of Toxicity of Chemicals" web server was used for toxicology assessment (19). See the Supporting Information for full experimental details.

### Cell migration and invasion studies

The wound healing assay was performed using two breast cancer cell lines, MDA-MB-231 and MCF-7, and two prostate cancer cell lines, PC-3 and DU-145, to evaluate the antimigratory capacity of the peptides. Cells were cultured in DMEM supplemented with 10% FBS, and sub-IC50 concentrations of the MIEN1-derived peptides and their analogs were prepared. The cells were grown to approximately 80% confluency in 12-well tissue culture plates, and a cell-free area was created by scraping the monolayer. The peptides were added, and the cells were incubated at 37 °C with 5% CO_2_ for 24 h. Wound closure was assessed by imaging the wounds at 0 and 24 h, and the wound areas were quantified using ImageJ software (https://imagej.net/ij/). In the transwell invasion assay, MDA-MB-231 cells were serum starved for 4 h followed by a pretreatment with increasing concentrations of each peptide in a 0.1% serum-containing DMEM for 24 h. The cells were then trypsinized, resuspended in same media for the second treatment of peptides, and counted. Next, 40,000 cells were resuspended in 0.1% serum containing DMEM and added to the upper chamber of Transwell plates (Corning Life Sciences) with a polyethylene terephthalate membrane (6.4 mm diameter, 8 μm pore size). The bottom chambers were filled with DMEM containing 10% FBS as a chemoattractant. The transwell plates were incubated at 37 °C with 5% CO_2_ for 24 h to allow the invasion of cells through the membrane. The invaded cells on the lower side of the membrane were fixed with 4% paraformaldehyde for 15 min, followed by a PBS wash. The membrane inserts were further fixed with ice-cold methanol for 20 min and stained with 0.25% (w/v) crystal violet. After incubation, the noninvasive cells on the upper surface of the membrane were gently removed using a cotton swab. Random fields were chosen for imaging, and the number of invaded cells was counted using ImageJ software. The results were expressed as the average number of invaded cells compared with the control.

### RT-PCR

Total RNA was extracted using the TRIzol reagent (Invitrogen). One microgram of total RNA was used for complementary DNA reverse transcription using the Superscript-III First Strand Synthesis kit (Invitrogen) following the manufacturer's protocol. All real-time PCR reactions were performed in duplicates in a 20 μl volume using SYBR Green Master Mix (Invitrogen) in a Realplex2 Mastercycler ep gradient S thermal cycler (Eppendorf). The PCR program followed for all the primers was an activation at 95 °C for 15 min, followed by 40 cycles of 95 °C for 30 s, 54 °C for 1 min, and 72 °C for 45 s. The reaction was finally put to hold at 4 °C. Subsequently, 18S was used as the reference gene for the mRNA levels. Relative change in gene expression was calculated according to the ΔΔCt method (53, 54).

### RNA-seq

RNA isolation was conducted using the RNeasy Mini Kit (Qiagen). The purification process included a 15-min DNase treatment step with the RNase-free DNase Set, ensuring the acquisition of DNA-free RNA. Triplicate samples of RNA were isolated, each exhibiting an RNA integrity number value exceeding 9. Subsequent library preparation used the Illumina TruSeq Stranded mRNA kit. Following the manufacturer's protocol, the prepared libraries underwent denaturation and dilution before sequencing on the NextSeq 550 platform with the NextSeq 550 High Output kit (150 cycles).To process the sequencing data, raw files were subjected to adapter trimming and quality filtering using Fastp (55). The reads were mapped to the hg38 reference genome using HISAT2 (56). For differential gene expression analysis, Deseq2 tool was used (57).

### Whole-cell lysate and subcellular fractionation for Western blot analysis

Western blot analysis was performed according to standard protocols to assess the effects of MIEN1-derived peptides and their analogs on MDA-MB-231 cells and DU-145 cells. Cell lysates were prepared using radioimmunoprecipitation assay buffer and quantified for protein content by bicinchoninic acid method. Subsequently, 40 μg of total protein was separated on a 4 to 12% precast polyacrylamide gel and transferred to a polyvinylidene fluoride membrane. Protein blocking with 5% bovine serum albumin was followed by incubation with primary antibodies specific to various proteins at 4 °C for 8 to 10 h. The membrane was then washed three times in Tris Buffered Saline with Tween 20 and incubated with the appropriate secondary antibody. Protein bands were visualized using the Alpha Innotech chemiluminescent detection system. In case of MDA-MB-231 cells, cytoplasmic and nuclear extracts were isolated to investigate the effects of peptides and/or EGF stimulation. Prior to exposure to the cells, the test peptides (90 μM) were incubated with EGF (10 ng/ml) in media at 4 ºC for 15 min. The untreated MDA-MB-231 cells received EGF alone at the same concentration. The isolation of cytoplasmic and nuclear fractions was performed using the Subcellular Protein Fractionation Kit for Cultured Cells from Thermo Fisher Scientific (cat #78840) following the manufacturer's guidelines. For Western blot experiments, 20 μg of purified cytoplasmic and nuclear fraction was used.

### Densitometry analysis

Densitometry analysis of Western blot data was conducted using the AlphaView-FluorChem HD2 software of the Alpha Innotech chemiluminescent detection system (https://www.cambridgescientific.com/product/alpha-innotech-fluorchem-hd2-gel-imaging-system). To quantify band intensities, a uniform rectangle was drawn around each band, and background subtraction was applied to determine the total pixel density for each lane. Further calculations, including the determination of the ratio of phosphorylated proteins to total proteins or other loading controls, were carried out using Microsoft Excel. The graphical representation of the data was generated using GraphPad Prism 9.4.0 software (https://www.graphpad.com/updates/prism-940-release-notes).

### Computational molecular modeling and CD studies for peptide-MIEN1 interaction

For the docking process involving the MIEN1 protein and the peptides LA3IK and RP-7, the Swiss Dock web server was used (58). CD spectra for all the peptides were recorded on a Jasco J-815 spectropolarimeter with/without MIEN1 protein. For experimental details, see the Supporting Information.

### Analytical LC-MS/MS

Levels of (D/L)-LA3IK and (D/L)-RP-7 for *in vitro* and *in vivo* pharmacology assays were monitored by liquid chromatography with tandem mass spectrometry (LC-MS/MS) using a Sciex 6500 QTRAP mass spectrometer coupled to a Shimadzu Prominence LC. The compounds were detected with the mass spectrometer in positive multiple reaction monitoring mode by following the precursor to fragment ion transition 655.3 to 411.3 (LA3IK), 838.6 to 640.4 (RP-7). An Agilent Poroshell 120 EC-C18 column (2.7 micron, 50 × 3.0 mm) was used for chromatography with the following conditions: Buffer A: dH_2_O + 10% acetonitrile, 0.1% formic acid and 2 mM ammonium acetate; Buffer B: dH_2_O + 90% acetonitrile, 0.1% formic acid and 2 mM ammonium acetate; 0 to 0.5 min 3% B, 0.5 to 3.0 min gradient to 100% B, 3.0 to 3.5 min hold 100% B, 3.5 to 3.51 min gradient to 3% B, 3.51 to 4.5 hold 3% B. Tolbutamide (transition 271.2.2 to 91.2) from Sigma-Aldrich was used as an internal standard (IS).

### Mouse liver microsome stability

Male Institute of Cancer Research/CD-1 mouse microsome fractions (lot 2110330) were purchased from XenoTech. Microsome protein (0.5 mg/ml) was placed in a glass screw cap tube; a 2 mM dimethyl sulfoxide stock of (D/L)-LA3IK and (D/L)-RP-7 were spiked into separate 50 mM Tris, pH 7.5 solution, and this was added to the microsome solution on ice. The final concentrations of D/L- LA3IK and RP-7 after addition of all reagents were 2 μM. An NADPH-regenerating system (1.7 mg/ml NADP, 7.8 mg/ml glucose-6-phosphate, 6 U/ml glucose-6-phosphate dehydrogenase in 2% w/v NaHCO_3_/10 mm MgCl_2_) was added for analysis of Phase I metabolism after heating both the regenerating solution and the sample tubes to 37 °C for 5 min in a 37 °C shaking water bath. The incubation was continued and at varying time points after addition of phase I cofactors, the reaction was stopped by the addition of 0.5 ml of acetonitrile containing IS and formic acid such that the final concentration of IS was 100 ng/ml and acid was 0.1%. Time 0 samples were stopped with the acetonitrile solution while still on ice prior to addition of the NADPH regenerating system and compound, which were subsequently added. The samples were incubated for 10 min at room temperature and then spun at 16,100*g* for 5 min in a microcentrifuge at 4 °C. The supernatant was analyzed by LC-MS/MS. The method described by McNaney *et al.* was used with modification for determination of metabolic stability half-life by substrate depletion (59). A “% remaining” value was used to assess the metabolic stability of a compound over time. The LC-MS/MS peak area of the incubated sample at each time point was divided by the LC-MS/MS peak area of the time 0 (T0) sample and multiplied by 100. The natural Log (LN) of the % remaining of compound was then plotted *versus* time (in min) and a linear regression curve was plotted going through y-intercept at LN (100). The half-life (T ½) was calculated as T ½ = 0.693/slope.

### Plasma stability

CD1 mouse plasma isolated using acidified citrate dextrose was purchased from BioIVT. Each peptide's dimethyl sulfoxide stock was then mixed into the plasma, resulting in a 2 μM final concentration. An aliquot was immediately collected for a zero time point and quenched with an equal volume of acetonitrile containing 0.2% formic acid and 200 ng/ml tolbutamide IS. The remaining plasma samples were incubated at 37 °C for up to 24 h. Samples were withdrawn at specific intervals and processed similarly to the zero time point. The supernatant obtained after vortexing and centrifugation was subjected to LC-MS/MS analysis as previously detailed. A control incubation was performed using 0.9% NaCl (saline).

### Pharmacokinetic studies

Pharmacokinetic studies were performed by dosing 6-week-old female CD1 mice (Charles River Laboratories) with D-LA3IK and D-RP-7 peptides by the indicated routes at 8 to 10 ml/kg formulated in 100% D5W. Animals were sacrificed in groups of three; blood was obtained by a submandibular puncture at each time point (0.08, 0.5, 1.5, 3, 6, 16, and 24 h post dose) into K_2_EDTA tubes and plasma was isolated by centrifugation at 9600*g* for 10 min. Subsequently, 50 μl of plasma was mixed with a 3× volume of acetonitrile containing formic acid and tolbutamide IS. The samples were vortexed for 15 s, incubated at room temperature for 10 min and spun twice at 16,100*g* 4 °C in a refrigerated microcentrifuge. The resulting supernatants were evaluated by LC-MS/MS as described above. Standard curves were generated using blank plasma (BioIVT) spiked with known concentrations of compound and processed as described above. The concentrations of drug in each time-point sample were quantified using Analyst software (Sciex, https://sciex.com/products/software/analyst-software#). Compounds were assumed to partition equally between red blood cells and plasma. A value of 3-fold above the signal obtained from blank plasma was designated the limit of detection. The limit of quantitation was defined as the lowest concentration at which back calculation yielded a concentration within 20% of theoretical. Terminal half-life (T ½), area under the concentration-time curve, volume of distribution at steady state (Vss), and clearance (Cl) values were calculated using the noncompartmental analysis tool of Phoenix WinNonlin 64 (version 8.3.3.33, Certara/Pharsight, https://www.certara.com/software/phoenix-winnonlin/).

### *In vivo* assays in an orthotopic mouse model of breast cancer

The *in vivo* experiments were carried out using female athymic nude mice aged 6 to 8 weeks (Charles River Laboratories). MDA-MB-231 cells (1.5 × 10^6^) were suspended in 50% Matrigel in DMEM high glucose (vol/vol) while maintaining cold temperatures, intended for injection into the mammary fat pad. The control group was administered an equivalent volume of Matrigel and media, excluding cells that served as vehicles. After a 10-day period, during which tumors became palpable, the mice were randomly distributed into three groups (5 mice/group). Subsequently, intraperitoneal injections of PBS, LA3IK, or RP-7 (30 mg/kg) were administered on alternating days for 30 days. Tumor volume measurements were performed on alternate days using a digital Vernier caliper. The modified ellipsoidal formula V=(W^2^ × L)/2 was used to calculate tumor volumes (V, tumor volume; W, tumor width; L, tumor length) in cubic millimeters (mm^3^). Mice were euthanized as per the approved protocols when tumors in the control group reached∼10% of body weight. The tumors were excised and the visceral organs like spleen, lung, liver, and kidneys were collected. Tumor masses of both control and the treated groups were weighed and documented.

### Study approval

The *in vivo* mouse models of breast cancer were performed under Institutional Animal Care and Committee-approved protocols at the University of North Texas Health Science Center. Studies to evaluate the pharmacokinetics of LA3IK and RP-7 were performed under Institutional Animal Care and Committee-approved protocols at UT Southwestern Medical Center.

### Statistical analysis

All data were analyzed for significance using GraphPad Prism 9.4.0 software. Two-tailed unpaired *t* test or one-way analysis of variance (ANOVA) were used to identify statistically significant differences. A *p*-value < 0.05 was regarded as statistically significant.

## Data availability

All data associated with this study are available within the article itself and its supporting information file. Raw data are available upon request from the corresponding authors. Sequencing data is available from the NCBI GEO database (accession number: GSE208776).

## Ethics approval

All animal studies were performed under Institutional Animal Care and Committee-approved protocol at the University of North Texas Health Science Center and Institutional Animal Care and Committee-approved protocols at UT Southwestern Medical Center.

## Supporting information

This article contains supporting information (19, 58, 60, 61, 62, 63).

## Conflict of interest

A. K. T. and J. K. V. report a patent relating to the work pending.
