# Supplementary material for: Short peptides based on the conserved regions of MIEN1 protein exhibit anticancer activity by targeting the MIEN1 signaling pathway
Source: J Biol Chem. 2024 Jan 23;300(3):105680. doi: 10.1016/j.jbc.2024.105680 (PMC10878790; doi:10.1016/j.jbc.2024.105680)
Supplement: Supporting Information [file mmc1.pdf]

Short Peptides based on the conserved regions of MIEN1 protein exhibit anti-cancer activity by targeting the MIEN1 Signaling Pathway

Running Title: MIEN1 protein-derived anti-cancer peptides.

Amit K. Tripathi1*, Priyanka P. Desai1, Antariksh Tyagi2, Jana B. Lampe1, Yogesh Srivastava3, Michael Donkor1, Harlan P. Jones1, Sergei V. Dzyuba4, Eric Crossley5, Noelle S. Williams5, Jamboor K. Vishwanatha1*.

1 Department of Microbiology, Immunology and Genetics, University of North Texas Health Science Center, Fort Worth, Texas 76107, USA.
2 Yale Center for Genome Analysis (YCGA), Yale School of Medicine.New Haven, CT 06510 USA.
3 Department of Genetics, University of Texas MD Anderson Cancer Center, Houston, TX, 77030, USA.
4 Department of Chemistry and Biochemistry, Texas Christian University, Fort Worth, TX 76129, USA.
5 Department of Biochemistry, University of Texas Southwestern Medical Center, Dallas, TX 75390, USA.
• Correspondence: amitkumar.tripathi@unthsc.edu & jamboor.vishwanatha@unthsc.edu

Supporting Information

- Full Experimental Method for Cell Viability Assay……S-2
- Full Experimental Method for Virtual Toxicology Assessment……S-2
- Full Experimental Method for Computational Molecular Modeling……S-3
- Full Experimental method for Circular dichroism (CD) spectra…… S-3
- Table S1. Additional MIEN1-derived Peptides Used in the Study……S-4
- Table S2. List of Primers Used in The Study for qPCR…… S-4
- HPLC and MS data for all the peptides……S-5
- Table S3. IC50 values of all the peptides in 4 different cell lines……S14
- Cell Viability data on tumorigenic & non-tumorigenic cell lines…… S-14
- Predicted LD50 and Toxicity Model Report of LA3IK & RP-7……S-15
- Light microscopy Visualizations of Wound Healing Assay of MIEN-1 derived peptides and its analogs …… ..S-16
- Wound Healing assay and Western Blot of D and L isomers of LA3IK and RP-7 on MDA-MB-231…… .S-17
- Wound Healing Assay using LA3IK and RP-7, along with their respective scrambled control analogs (LA3IK SCR and RP-7 SCR), on two breast and prostate cancer cell lines……S-17
- Circular Dichroism and Docking Experiments with LA3IK and RP-7…… S-18
- Molecular Docking Results of MIEN1protein with LA3IK and RP-7……S-18
- Full-length Western Blot images …… S-19
- References……S-23

Full Experimental Method for Cell Viability Assay:

Cell viability of murine NIH-3T3 cells, as well as MDA-MB-231, MCF-7, PC-3, and DU-145 cells, was assessed through the MTT assay as described previously(1). Approximately 5000 cells per well were seeded in a 96-well plate and allowed to incubate overnight for attachment. Following attachment, the growth medium was replaced with a fresh medium containing serial dilutions of peptides. The cells were then incubated for 48 hours in a 5% CO2 incubator. After incubation, 10 µl of a 5 mg/mL MTT solution was introduced to each well and left to incubate for an additional 2 hours. Subsequently, the entire medium, along with the MTT solution, was aspirated, and the remaining formazan crystals were dissolved in 200 µl of DMSO. The absorbance was measured at 595 nm using a Biotek Synergy 2 Plate Reader.

Full Experimental Method for Virtual Toxicology Assessment:

ProTox-II "Prediction of Toxicity of Chemicals" web server was used for toxicology assessment. The viability of the peptide-treated cells was determined with respect to the control cells of 100% viability, and IC50 for all five cell types was determined. For the toxicological assessment, the peptide SMILES sequences were generated through an online "convert" tool designed to facilitate the transformation between diverse molecular formats (accessible at https://datascience.unm.edu/tomcat/biocomp/convert). Following this, a comprehensive toxicological investigation for bioactive peptides LA3IK and RP-7 was conducted utilizing the ProTox-II "Prediction of Toxicity of Chemicals" web server, designed for the prediction of toxicities associated with small molecules. This platform incorporates distinct models optimized for predicting diverse toxicity endpoints, encompassing acute toxicity, hepatotoxicity, cytotoxicity, carcinogenicity, mutagenicity, immunotoxicity, as well as adverse outcomes in Tox21 pathways and toxicity targets(2)

Full Experimental Method for Computational Molecular Modeling:

The peptides and the MIEN1 protein were subjected to an energy minimization procedure using the UCSF ChimeraX software. The minimization process consisted of two steps: initially, 1000 iterations of the steepest descent method were applied, followed by 500 iterations of the conjugate gradient algorithm, using a step size of 0.002 Å. The protein models were minimized utilizing the AMBER FF14SB force field(3). Molecular structures, highlighting the specific residues were visually depicted using UCSF ChimeraX.For the docking process involving the MIEN1 protein and the peptides LA3IK and RP-7, the Swiss Dock web server was employed(4) , which uses CHARMM energies to rank biomolecules in the docking studies (5). Clusters of MIEN1 and peptide structures were examined using an implicit solvation model (6). The interpretation of the docking results and the creation of model figures were accomplished using UCSF Chimera version 1.15 and the analysis and visualization of docking energies were carried out using GraphPad Prism 9.4.0.

Full Experimental method for Circular dichroism (CD) spectra:

Circular dichroism (CD) spectra for all the peptides were recorded on a Jasco J-815 spectropolarimeter with/without MIEN1 protein. The appropriate concentration of peptide or protein stock solution was added in PBS (pH 7.4) and gently mixed using a micropipette in a microcentrifuge tube, and subsequently transferred into a 0.1 cm quartz cell for CD measurements. Spectra were acquired at room temperature and 1 nm resolution with a scan rate of 50 or 100 nm/min. 2-4 scans were acquired and averaged for each sample. Raw data were manipulated by subtraction of appropriate background spectra, followed by smoothing using manufacturer-provided software. Specifically, the CD spectra of the peptides (without MIEN1) were subtracted from the CD spectra of the Peptide and MIEN1 to show any conformational changes after the addition of the MIEN1 protein. Data are expressed as mean residue ellipticity (MRE).

Table S1. Additional MIEN1-derived Peptides Used in the Study

Sl No	Peptide Name	Peptide Sequence	Activity
1	YC-5	Ac-YCEPC-NH2	No Anti-Cancer Activity
2	LI-7	Ac-LIEAIRR-NH2	No Anti-Cancer Activity

Table S2. List of Primers Used in The Study for qPCR

Target gene	Forward primer (5'>3')	Reverse primer (5'>3')
**18S**	GTA ACC CGT TGA ACC CCA TT	CCA TCC AAT CGG TAG TAG CG
**MMP9**	TTG ACA GCG ACA AGA AGT GG	GCC ATT CAC GTC GTC CTT AT
**E-CADERIN**	TGG CGT CTG TAG GAA GGC A	GGC TCT TTG ACC ACC GCT CT
**N-CADHERIN**	ACC AGG ACT ATG ACT TGA GCC	GGC GTG GAT GGG TCT TTC A
**SNAIL**	TCG GAA GCC TAA CTA CAG CGA	AGA TGA GCA TTG GCA GCG AG
**SLUG**	AAG CAT TTC AAC GCC TCC AAA	GGA TCT CTG GTT GTG GTA TGA CA
**ZEB1**	TGT GGT AGA AAC AAA TTC AGA TTC	GCC CTT CCT TTC CTG TGT CA
**IL-8**	GTG CAG TTT TGC CAA GGA GT	TTA TGA ATT CTC AGC CCT CTT CAA AAA CTT CTC
**MIEN1**	CAG TGC TGT GAA GGA GCA GT	GAC GGC TGT TGG TGA TCT TT

Figure S1:

LA-6

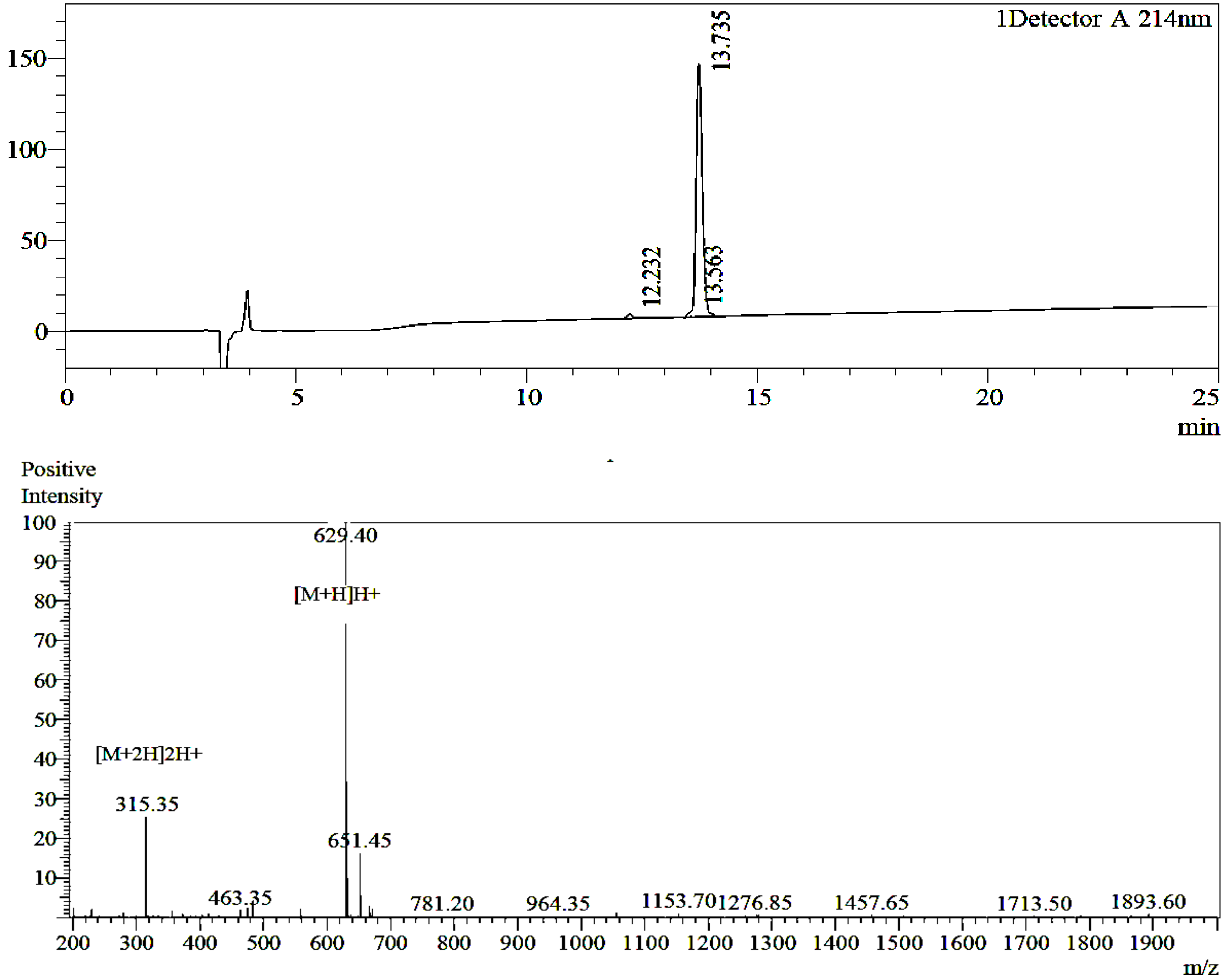

LA3IK

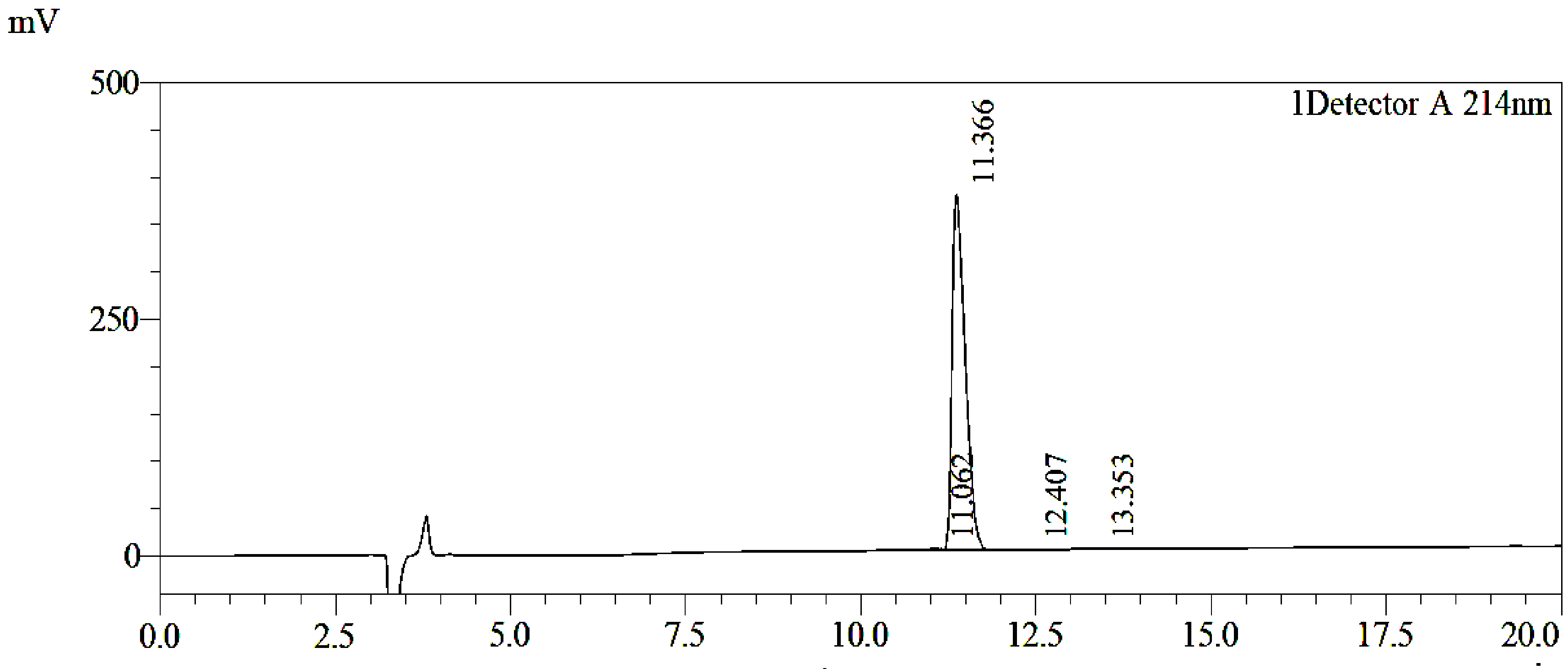
mV
500
250
0
1Detector A 214nm
11.366
11.062
12.407
13.353
0.0
2.5
5.0
7.5
10.0
12.5
15.0
17.5
20.0

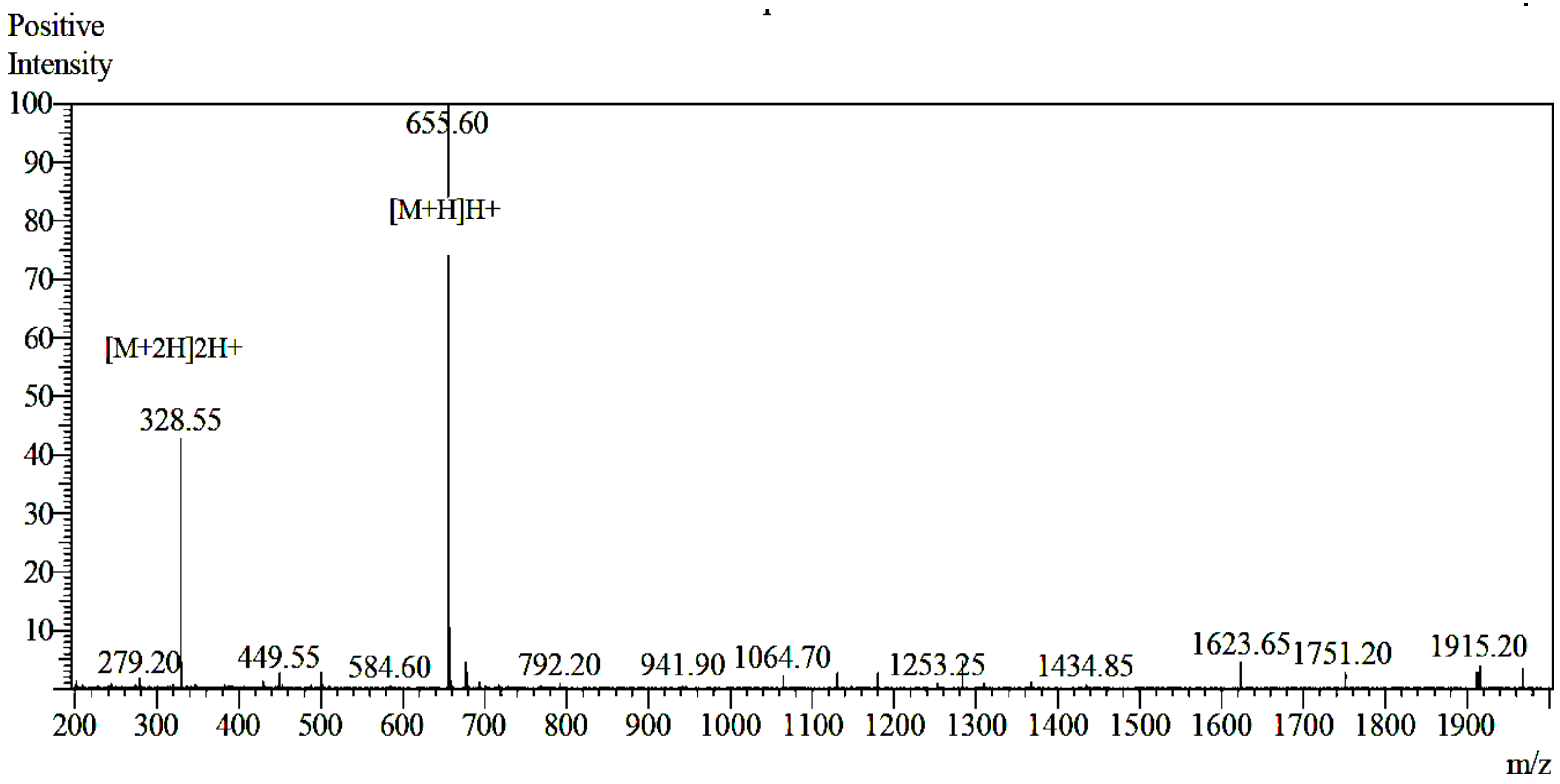
Positive Intensity
100
90
80
70
60
50
40
30
20
10
655.60
[M+H]H+
[M+2H]2H+
328.55
279.20
449.55
584.60
792.20
941.90
1064.70
1253.25
1434.85
1623.65
1751.20
1915.20
200 300 400 500 600 700 800 900 1000 1100 1200 1300 1400 1500 1600 1700 1800 1900
m/z

SR-8

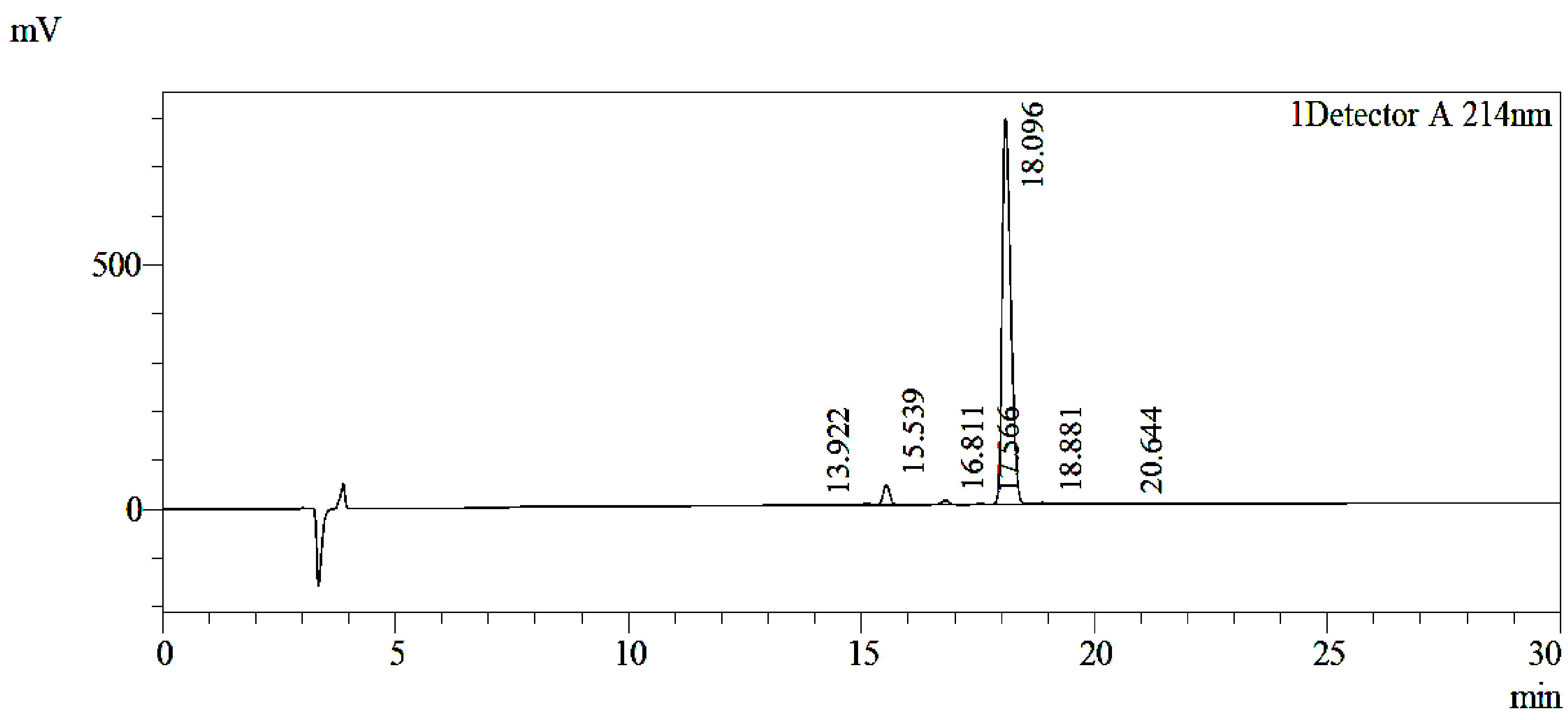
mV
1Detector A 214nm
500
0
18.096
13.922
15.539
16.811
17.566
18.881
20.644
0
5
10
15
20
25
30
min

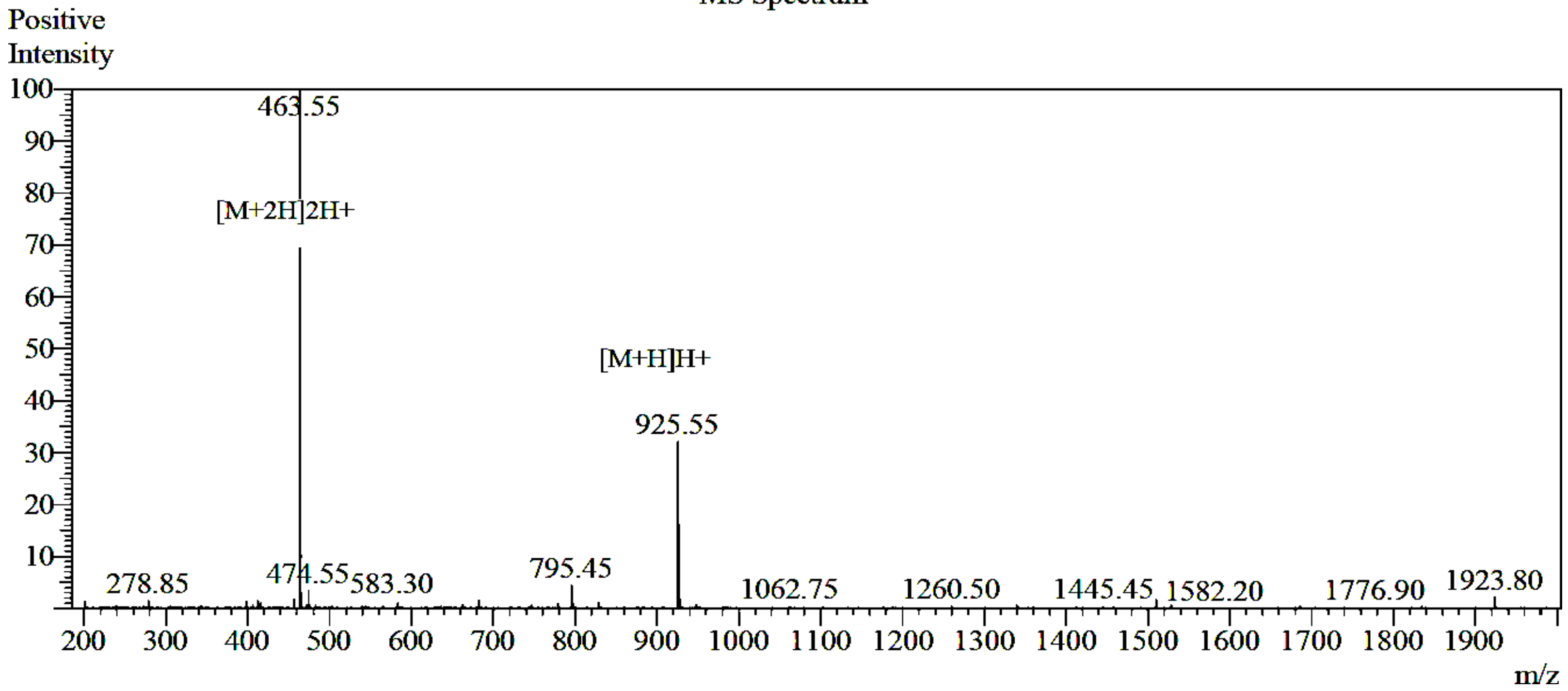
MS Spectrum
Positive Intensity
463.55
[M+2H]2H+
[M+H]H+
925.55
278.85
474.55
583.30
795.45
1062.75
1260.50
1445.45
1582.20
1776.90
1923.80
m/z

CR-8

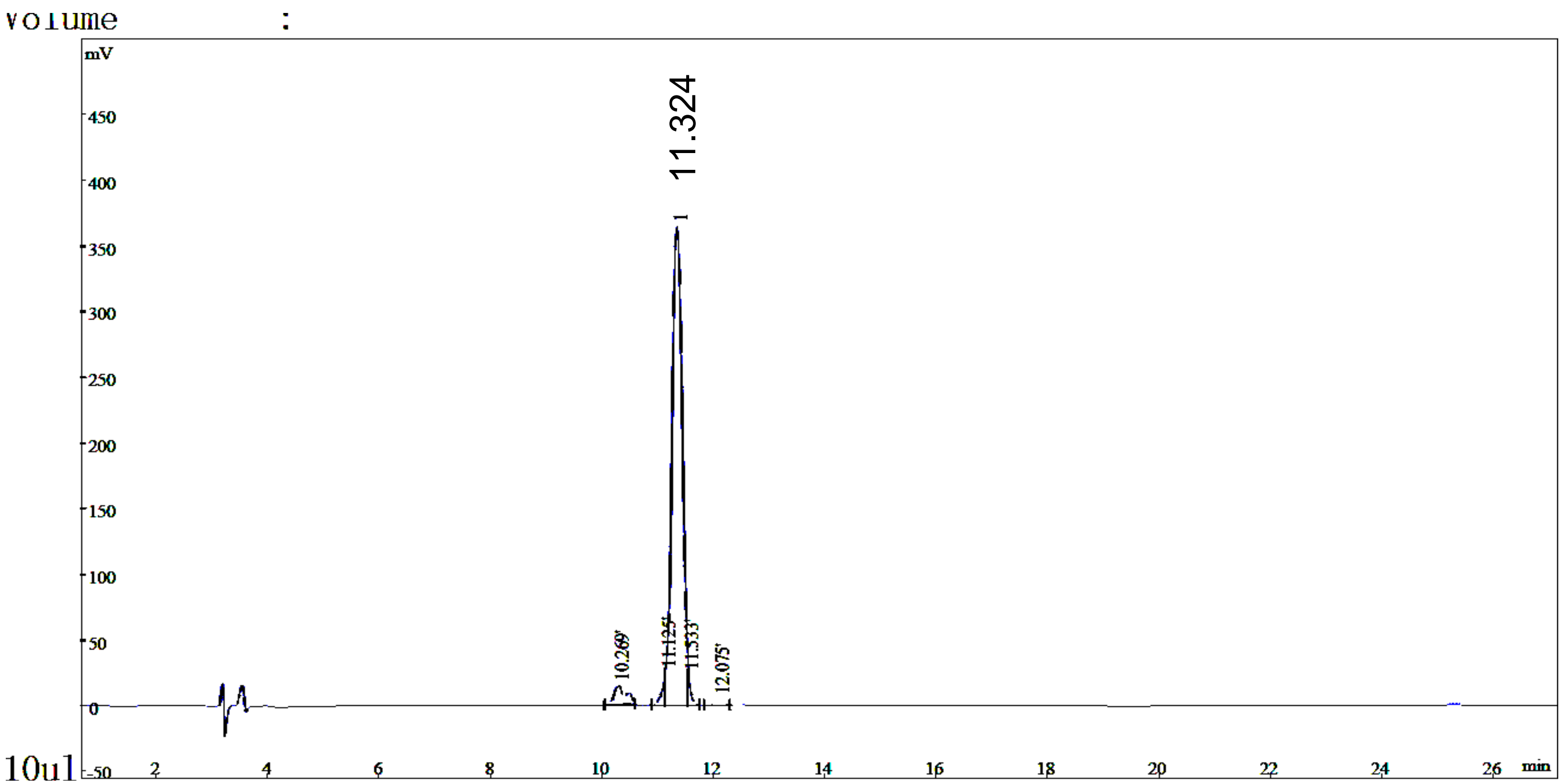
Volume :
mV
11.324
10.269'
11.125'
11.533'
12.075'
10ul
min

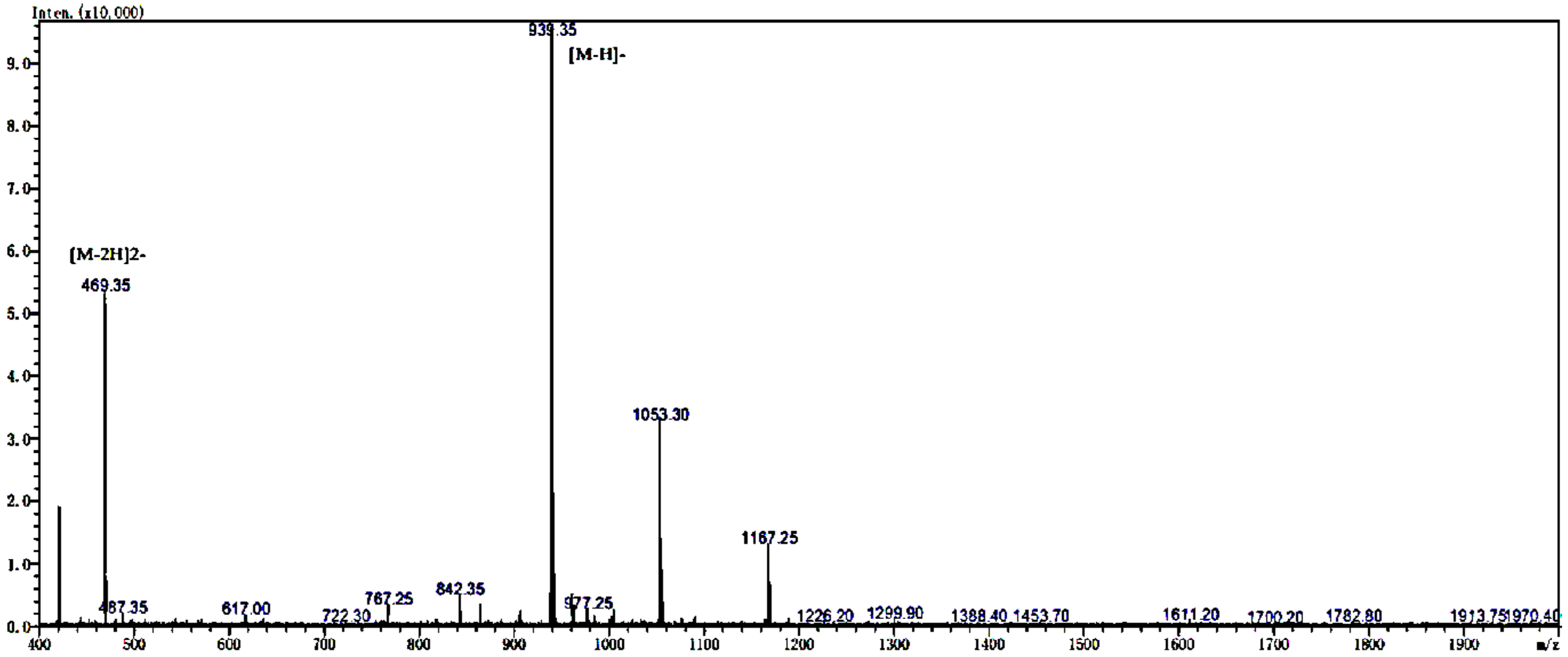
Inten. (x10,000)
939.35
[M-H]-
[M-2H]2-
469.35
1053.30
1167.25
487.35
617.00
722.30
767.25
842.35
977.25
1226.20
1299.90
1388.40
1453.70
1611.20
1700.20
1782.80
1913.75
1970.40
m/z

RP-7

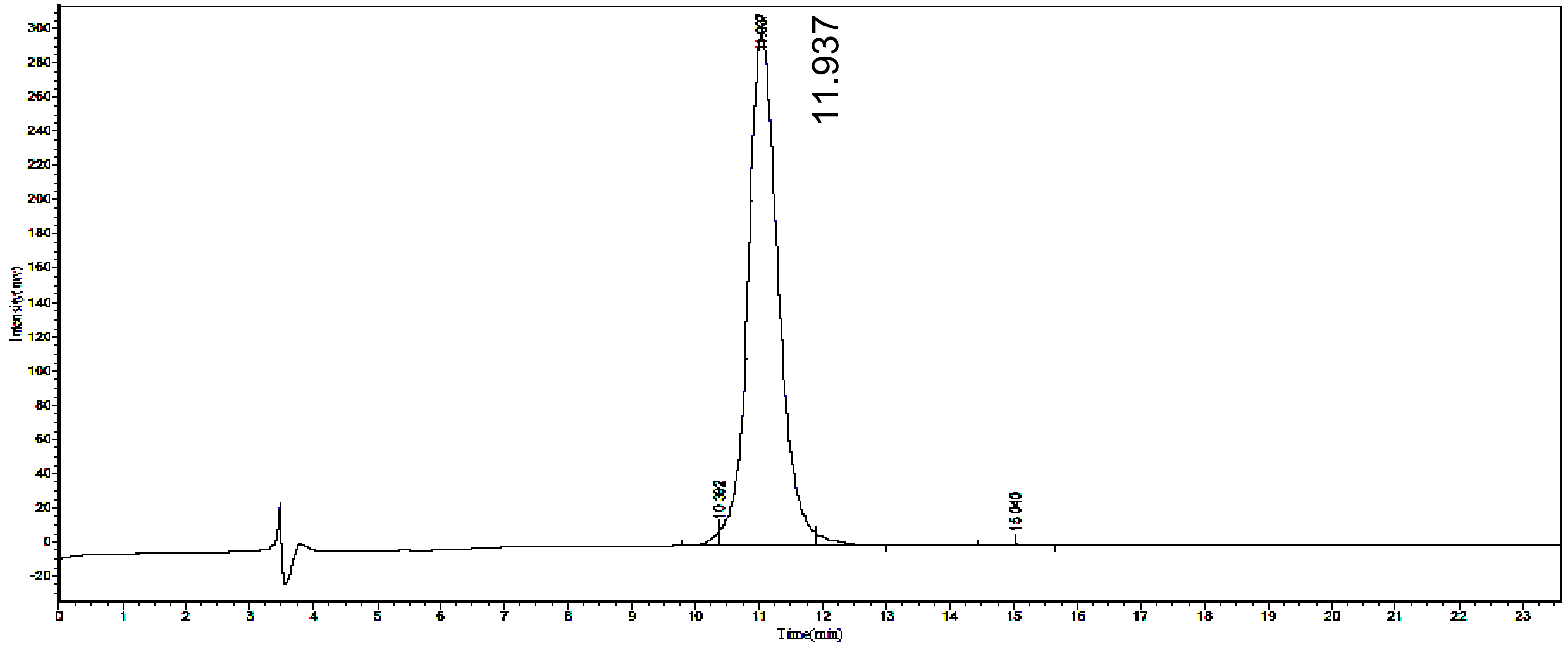

11.937
Intensity(mv)
Time(min)

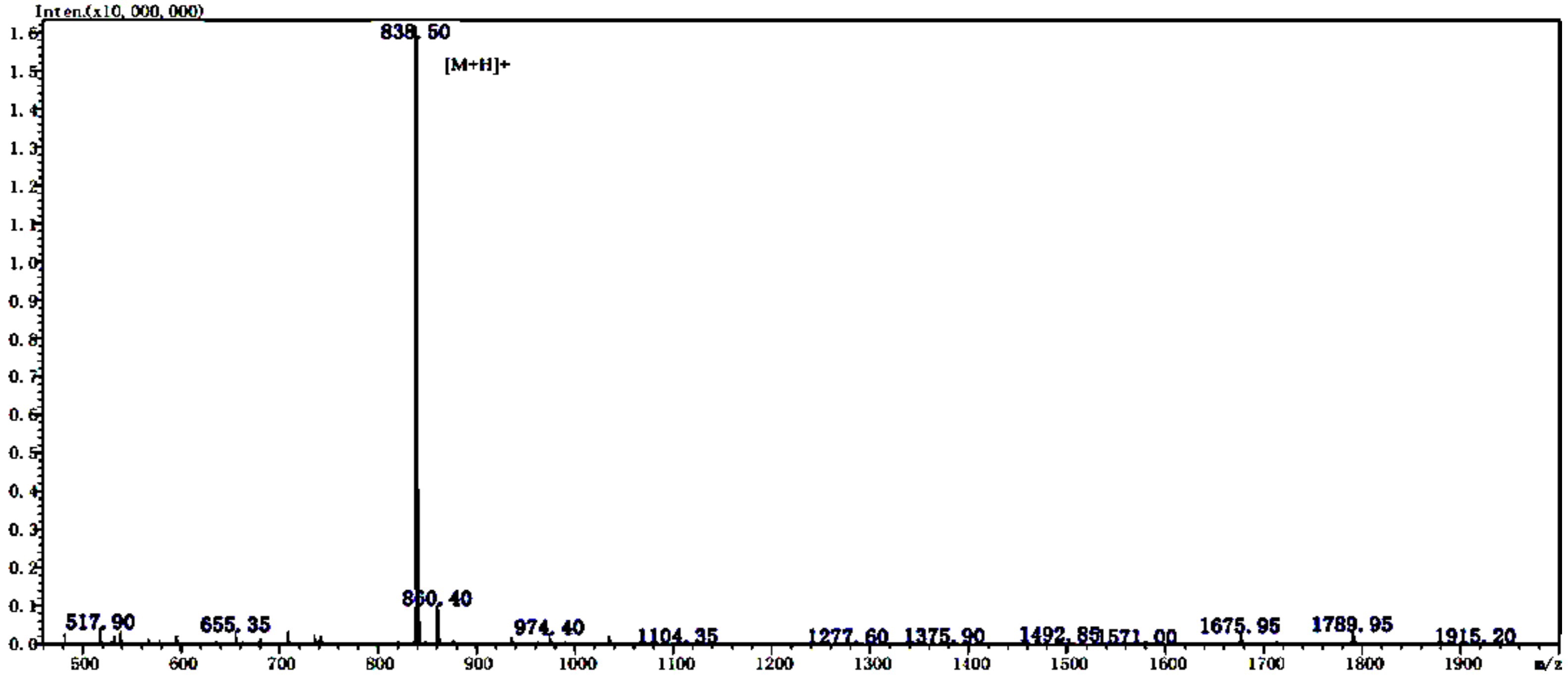

Inten.(x10, 000, 000)
838.50
[M+H]+
860.40
517.90
655.35
974.40
1104.35
1277.60
1375.90
1492.85
1571.00
1675.95
1789.95
1915.20
m/z

D-LA3IK

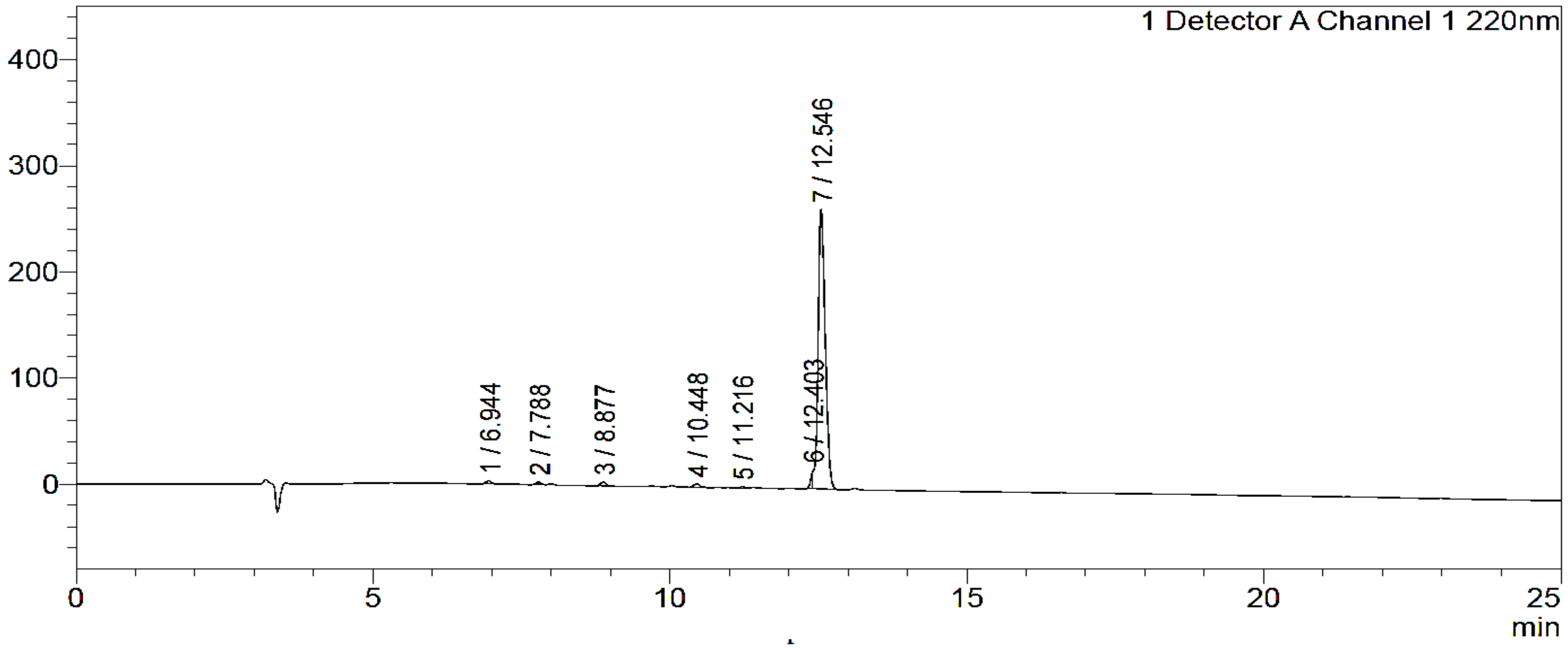
1 Detector A Channel 1 220nm
400
300
200
100
0
1 / 6.944
2 / 7.788
3 / 8.877
4 / 10.448
5 / 11.216
6 / 12.403
7 / 12.546
0
5
10
15
20
25
min

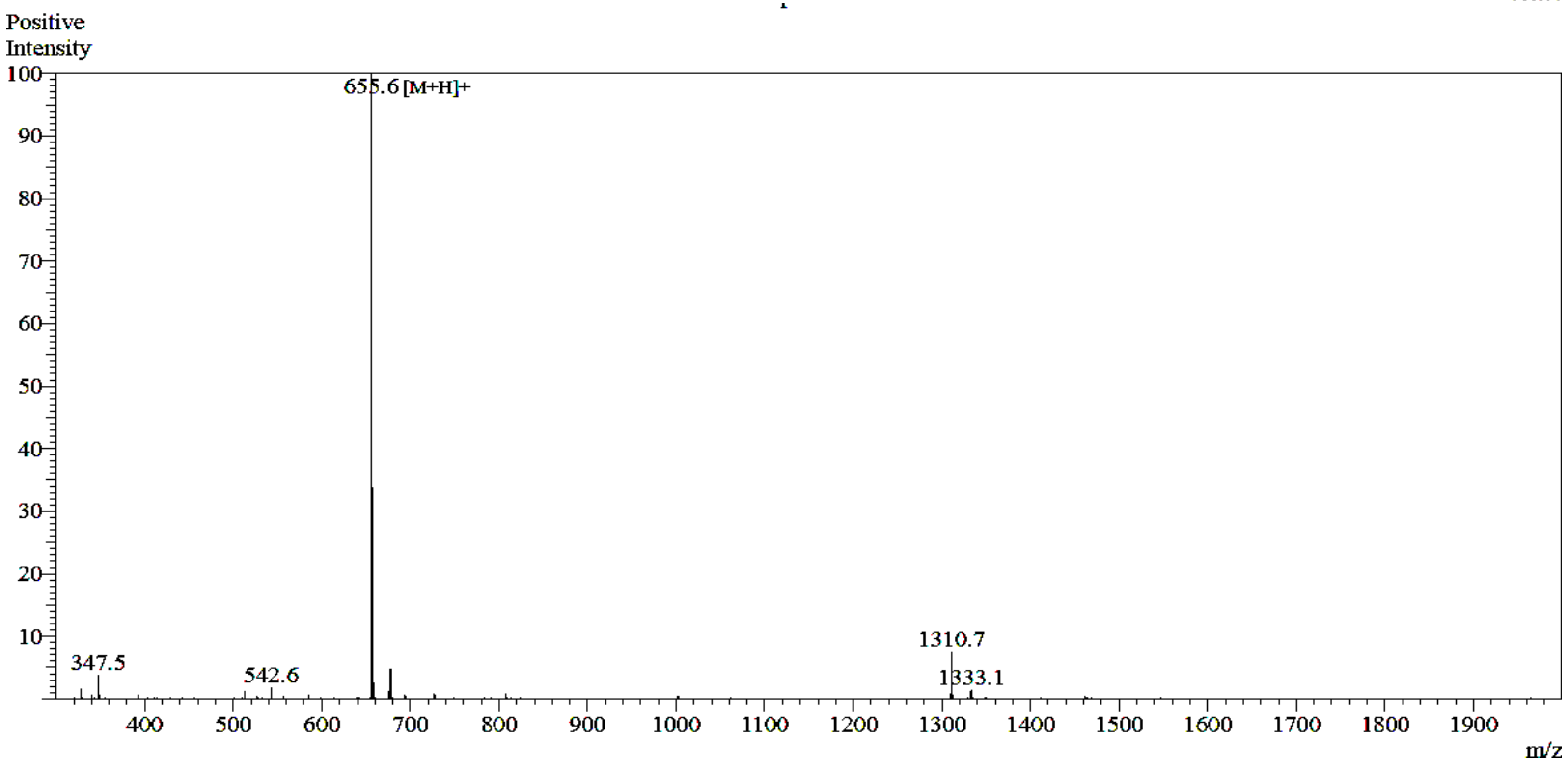
Positive
Intensity
100
90
80
70
60
50
40
30
20
10
655.6 [M+H]+
347.5
542.6
1310.7
1333.1
400
500
600
700
800
900
1000
1100
1200
1300
1400
1500
1600
1700
1800
1900
m/z

D-RP-7

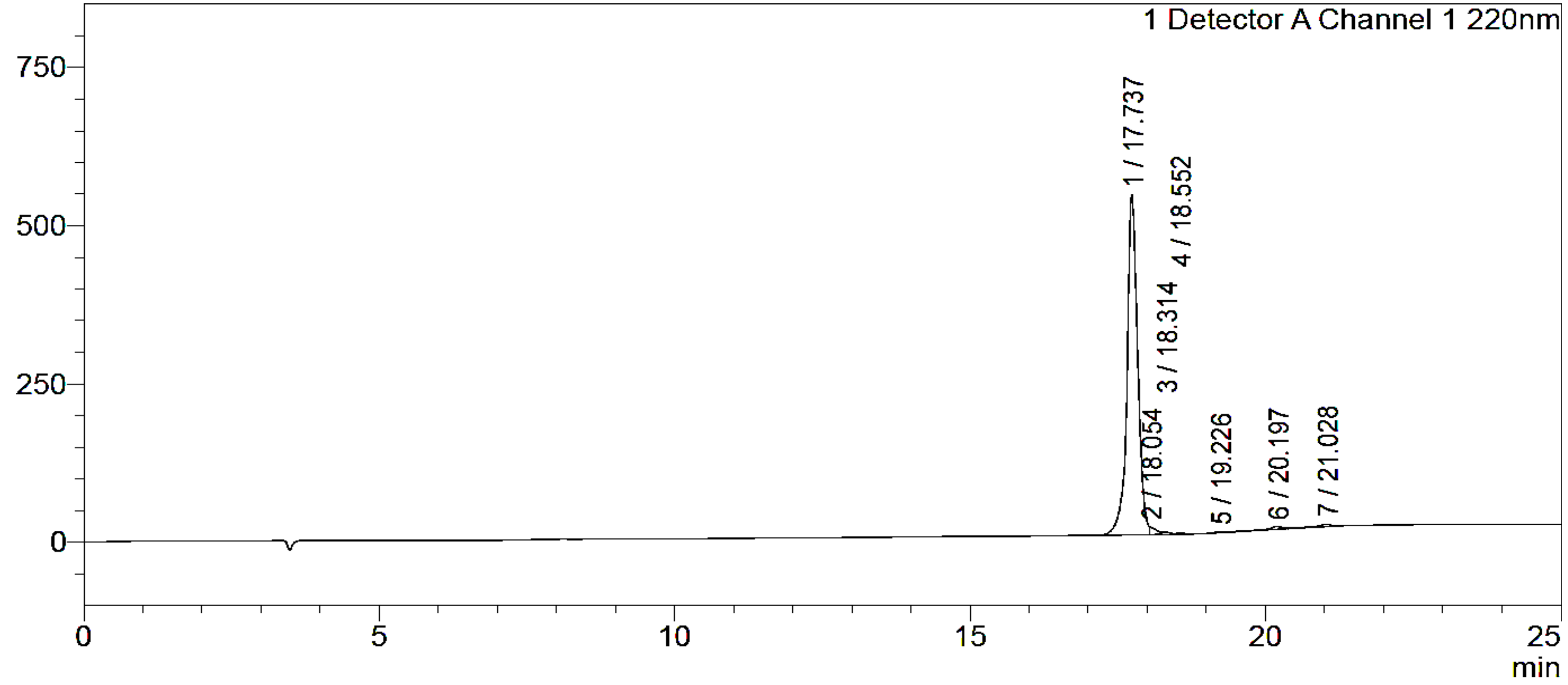
1 Detector A Channel 1 220nm
750
500
250
0
1 / 17.737
2 / 18.054
3 / 18.314
4 / 18.552
5 / 19.226
6 / 20.197
7 / 21.028
0
5
10
15
20
25
min

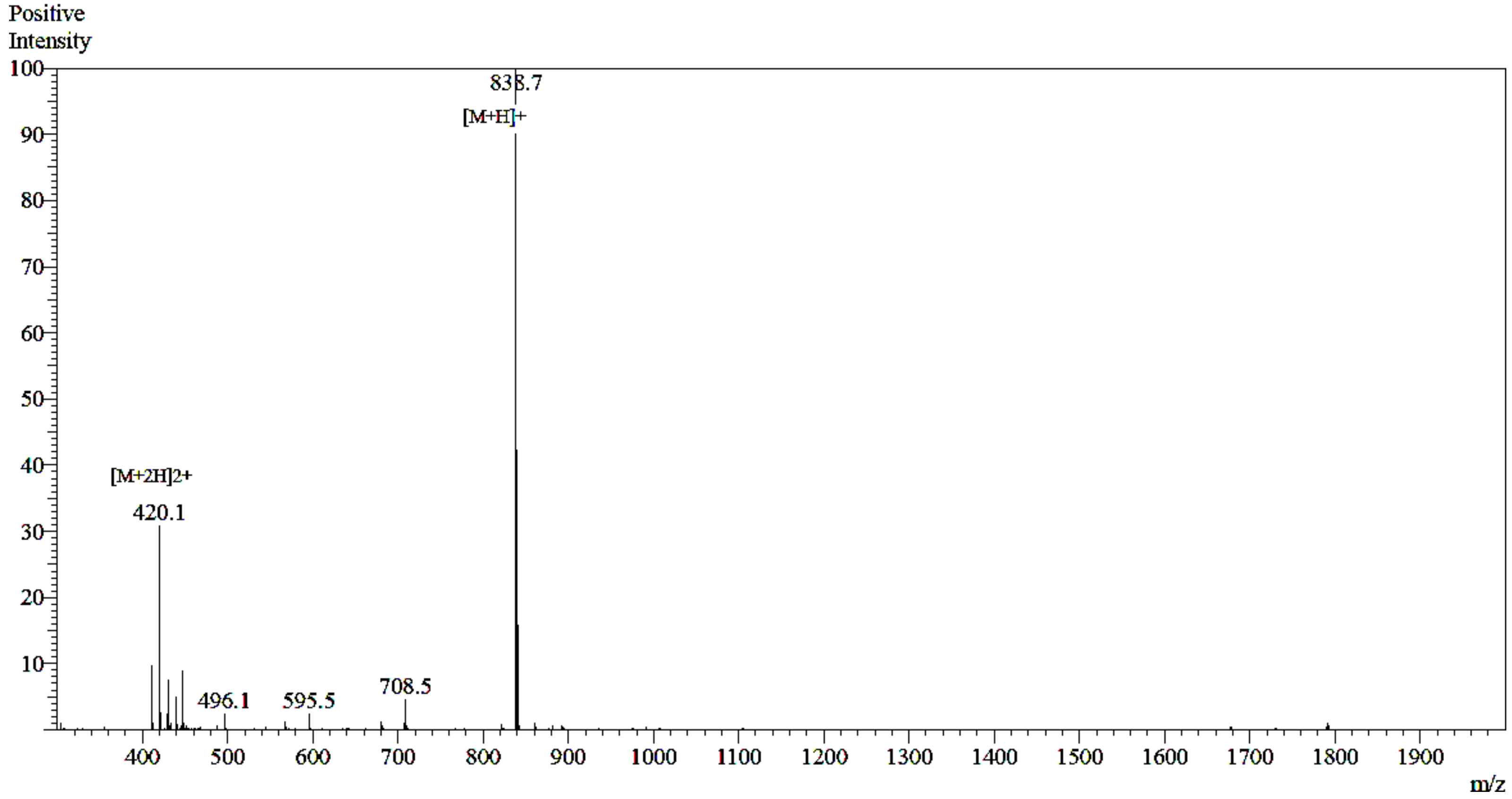
Positive
Intensity
100
90
80
70
60
50
40
30
20
10
838.7
[M+H]+
[M+2H]2+
420.1
496.1
595.5
708.5
400
500
600
700
800
900
1000
1100
1200
1300
1400
1500
1600
1700
1800
1900
m/z

LA3IK SCR

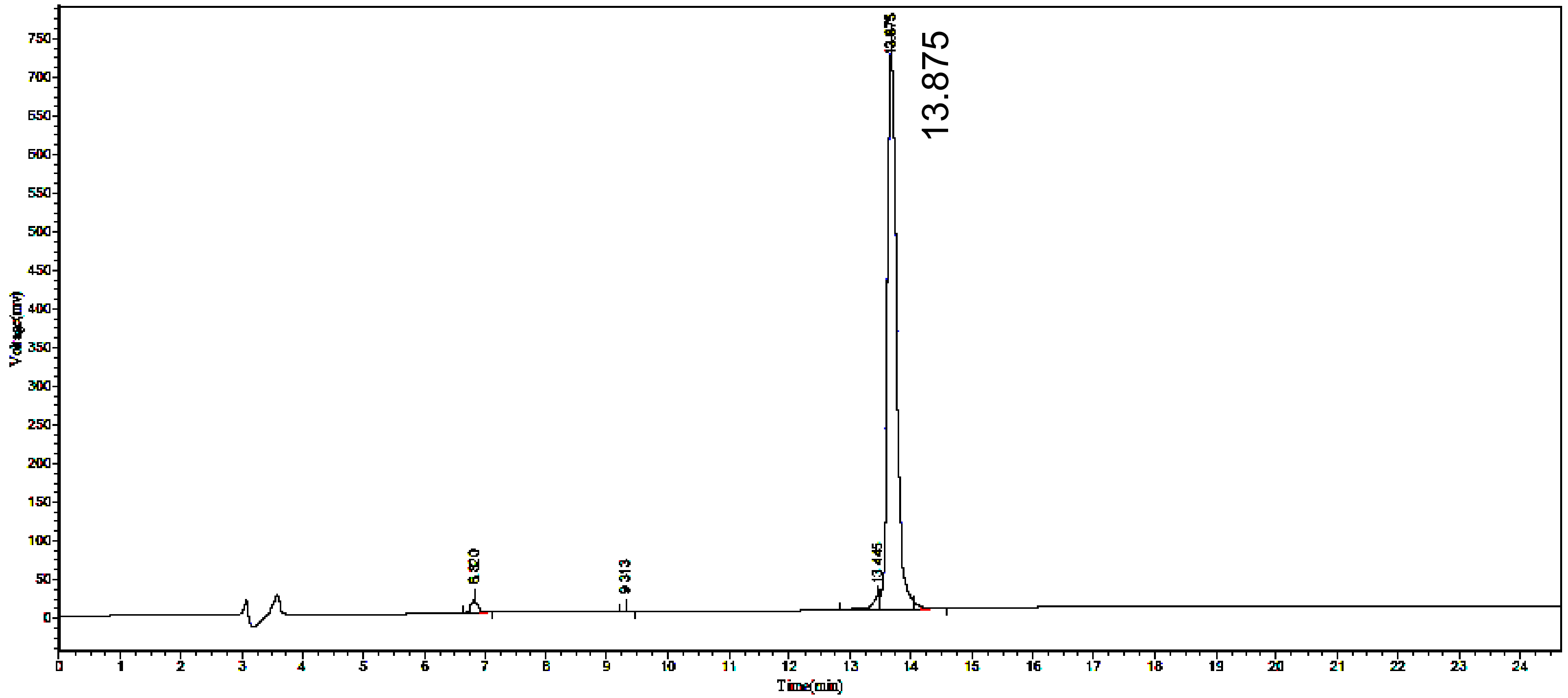

13.875
Voltage(mv)
Time(min)
6.820
9.313
13.445

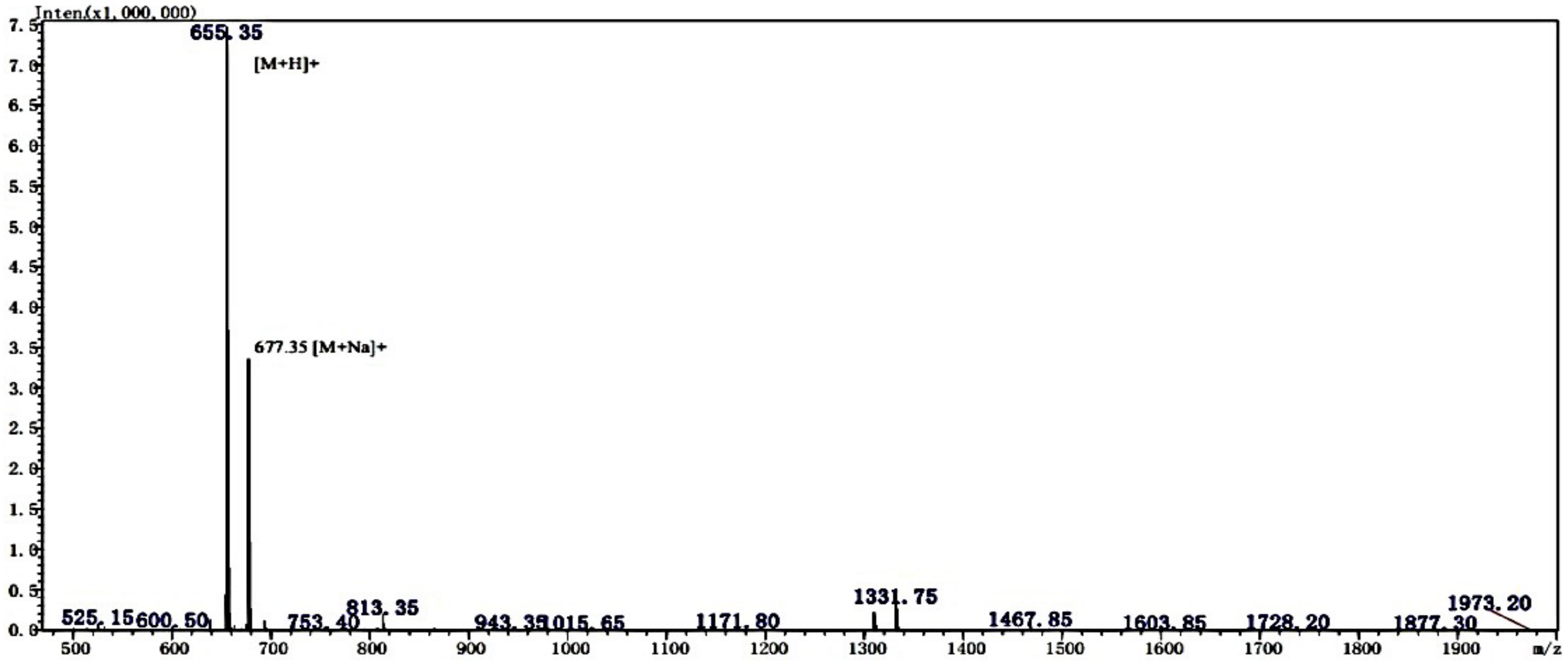

Inten.(x1,000,000)
655.35
[M+H]+
677.35 [M+Na]+
525.15
600.50
753.40
813.35
943.35
1015.65
1171.80
1331.75
1467.85
1603.85
1728.20
1877.30
1973.20
m/z

RP-7 SCR

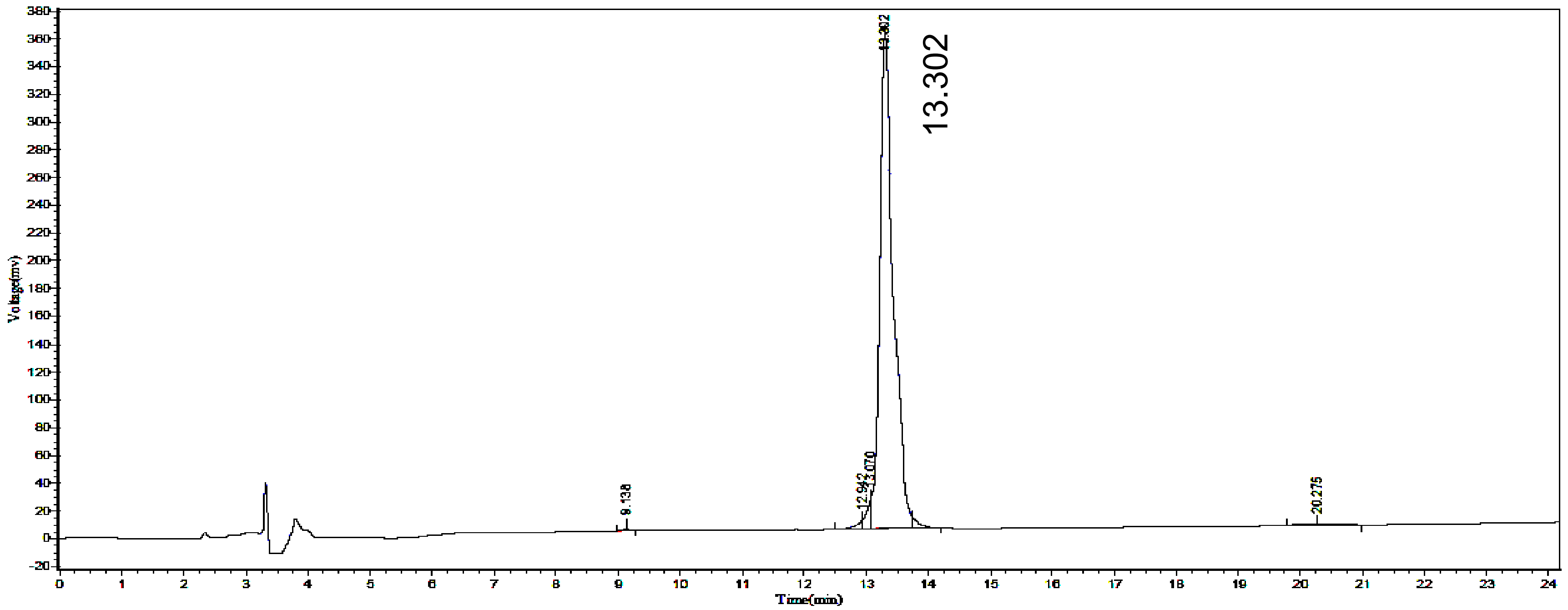
13.302
Voltage(mv)
Time(min)
9.138
12.942
13.070
20.275

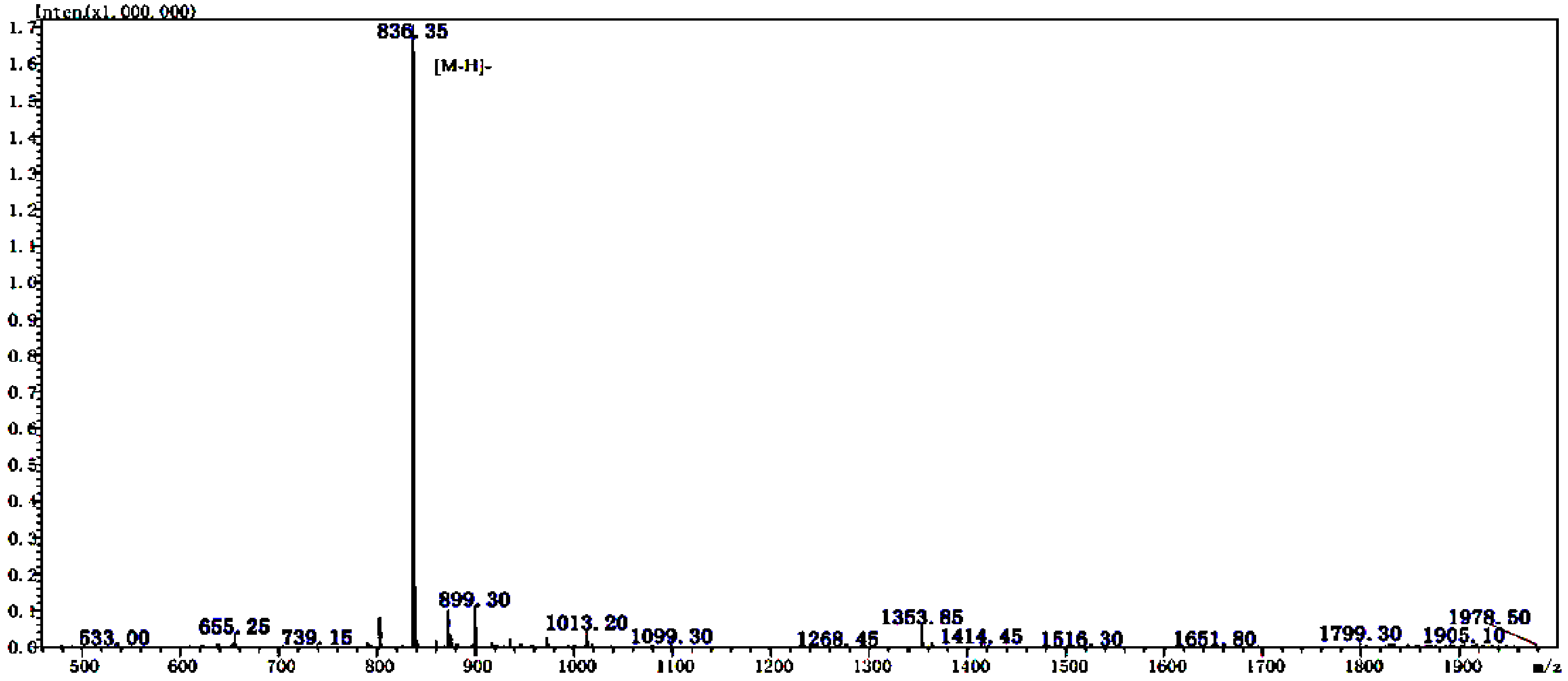
Inten.(x1,000,000)
836.35
[M-H]-
899.30
1013.20
1099.30
1268.45
1353.85
1414.45
1516.30
1651.80
1799.30
1905.10
1978.50
633.00
655.25
739.15
m/z

Figure S2:

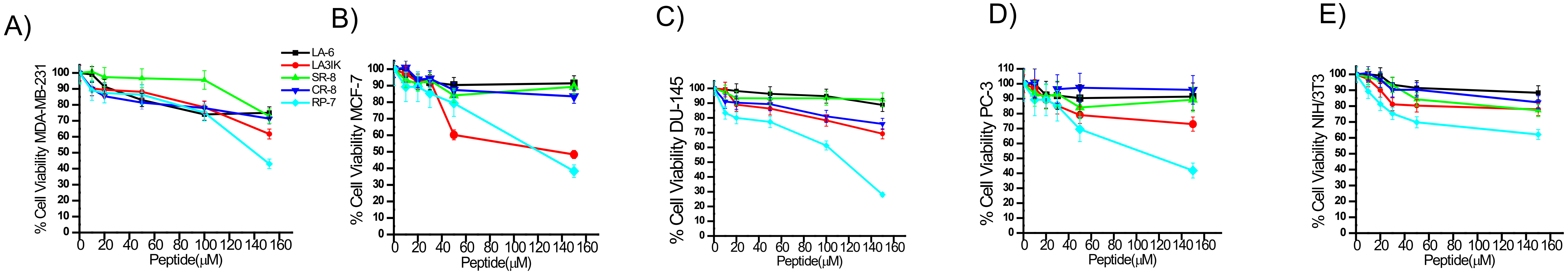

Table S3: IC50 values of all the peptides in 5 different cell lines

Peptide Name	MDA-MB-231 (IC50 μM)	MCF-7(IC50 μM)	PC-3(IC50 μM)	DU-145(IC50 μM)	NIH-3T3(IC50 μM)
LA-6	>600	>600	>600	>600	>600
LA3IK	223.6±5.62	90.23±7.32	321.4±11.79	335.7±3.09	>600
SR-8	528.28±4.57	141.32±12.91	>600	>600	>600
CR-8	>600	503.23±8.34	>600	>600	>600
RP-7	139.03±8.41	110.12±4.23	113±8.02	120.72±7.08	221±6.23
D- LA3IK	197.72±5.42	91.2±8.21	98.71±6.17	89.11±8.12	>600
D- RP-7	95±7.71	90±10.31	101±5.73	121.59±6.56	210±8.41
LA3IK-SCR	243±8.16	133±9.52	319±13.64	351.76±7.18	>600
RP7 SCR	156±8.15	150±10.32	128±11.65	137±5.98	>600

Figure S3:

LA3IK Toxicology Prediction

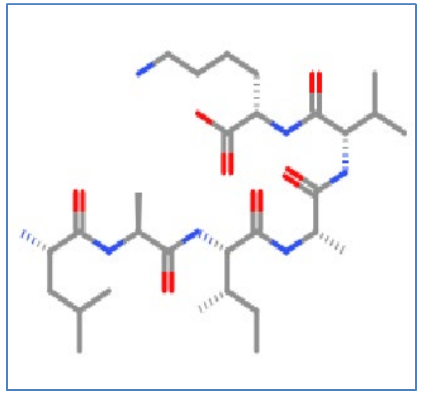

Predicted LD50: 3000mg/kg

Predicted Toxicity Class: 5

1 2 3 4 5 6

Average similarity: 82.63%

Prediction accuracy: 70.97%

20% 40% 60% 80%

Classification	Target	Shorthand	Prediction	Probability
Organ toxicity	Hepatotoxicity	dili	Inactive	0.87
Toxicity end points	Carcinogenicity	carcino	Inactive	0.62
Toxicity end points	Immunotoxicity	immuno	Inactive	0.99
Toxicity end points	Mutagenicity	mutagen	Inactive	0.87
Toxicity end points	Cytotoxicity	cyto	Inactive	0.64
Tox21-Nuclear receptor signalling pathways	Aryl hydrocarbon Receptor (AhR)	nr_ahr	Inactive	0.99
Tox21-Nuclear receptor signalling pathways	Androgen Receptor (AR)	nr_ar	Inactive	0.90
Tox21-Nuclear receptor signalling pathways	Androgen Receptor Ligand Binding Domain (AR-LBD)	nr_ar_lbd	Inactive	0.95
Tox21-Nuclear receptor signalling pathways	Aromatase	nr_aromatase	Inactive	0.99
Tox21-Nuclear receptor signalling pathways	Estrogen Receptor Alpha (ER)	nr_er	Inactive	0.86
Tox21-Nuclear receptor signalling pathways	Estrogen Receptor Ligand Binding Domain (ER-LBD)	nr_er_lbd	Inactive	0.96
Tox21-Nuclear receptor signalling pathways	Peroxisome Proliferator Activated Receptor Gamma (PPAR-Gamma)	nr_ppar_gamma	Inactive	0.97
Tox21-Stress response pathways	Nuclear factor (erythroid-derived 2)-like 2/antioxidant responsive element (nrf2/ARE)	sr_are	Inactive	0.98
Tox21-Stress response pathways	Heat shock factor response element (HSE)	sr_hse	Inactive	0.98
Tox21-Stress response pathways	Mitochondrial Membrane Potential (MMP)	sr_mmp	Inactive	0.99
Tox21-Stress response pathways	Phosphoprotein (Tumor Supressor) p53	sr_p53	Inactive	0.99
Tox21-Stress response pathways	ATPase family AAA domain-containing protein 5 (ATAD5)	sr_atad5	Inactive	0.99

RP-7 Toxicology Prediction

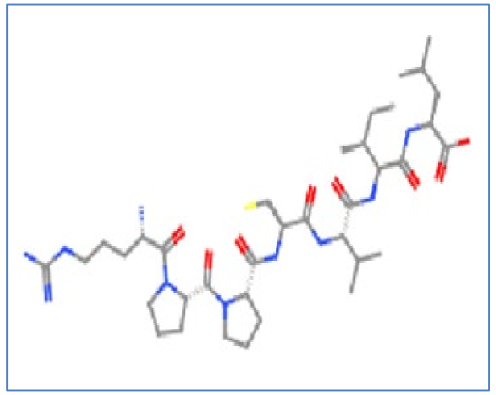

Predicted LD50: 3000mg/kg

Predicted Toxicity Class: 5

1 2 3 4 5 6

Average similarity: 70.21%

Prediction accuracy: 69.26%

20% 40% 60% 80%

Classification	Target	Shorthand	Prediction	Probability
Organ toxicity	Hepatotoxicity	dili	Inactive	0.92
Toxicity end points	Carcinogenicity	carcino	Inactive	0.59
Toxicity end points	Immunotoxicity	immuno	Inactive	0.99
Toxicity end points	Mutagenicity	mutagen	Inactive	0.74
Toxicity end points	Cytotoxicity	cyto	Inactive	0.78
Tox21-Nuclear receptor signalling pathways	Aryl hydrocarbon Receptor (AhR)	nr_ahr	Inactive	0.98
Tox21-Nuclear receptor signalling pathways	Androgen Receptor (AR)	nr_ar	Inactive	0.96
Tox21-Nuclear receptor signalling pathways	Androgen Receptor Ligand Binding Domain (AR-LBD)	nr_ar_lbd	Inactive	0.98
Tox21-Nuclear receptor signalling pathways	Aromatase	nr_aromatase	Inactive	0.98
Tox21-Nuclear receptor signalling pathways	Estrogen Receptor Alpha (ER)	nr_er	Inactive	0.88
Tox21-Nuclear receptor signalling pathways	Estrogen Receptor Ligand Binding Domain (ER-LBD)	nr_er_lbd	Inactive	0.96
Tox21-Nuclear receptor signalling pathways	Peroxisome Proliferator Activated Receptor Gamma (PPAR-Gamma)	nr_ppar_gamma	Inactive	0.96
Tox21-Stress response pathways	Nuclear factor (erythroid-derived 2)-like 2/antioxidant responsive element (nrf2/ARE)	sr_are	Inactive	0.97
Tox21-Stress response pathways	Heat shock factor response element (HSE)	sr_hse	Inactive	0.97
Tox21-Stress response pathways	Mitochondrial Membrane Potential (MMP)	sr_mmp	Inactive	0.96
Tox21-Stress response pathways	Phosphoprotein (Tumor Supressor) p53	sr_p53	Inactive	0.96
Tox21-Stress response pathways	ATPase family AAA domain-containing protein 5 (ATAD5)	sr_atad5	Inactive	0.99

Figure S4:

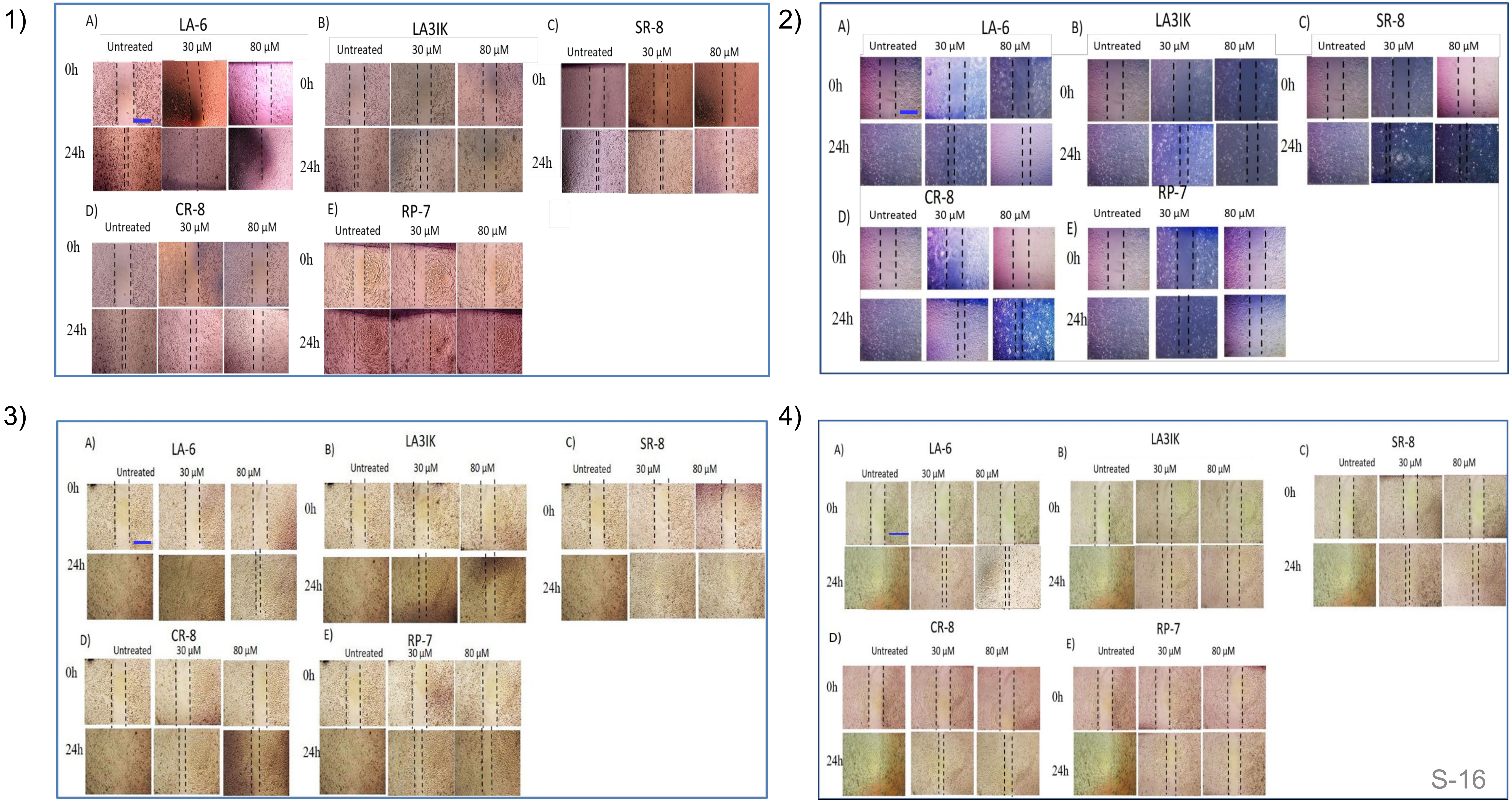
1)
A) LA-6
B) LA3IK
C) SR-8
D) CR-8
E) RP-7
Untreated
30 µM
80 µM
0h
24h
2)
A) LA-6
B) LA3IK
C) SR-8
D) CR-8
E) RP-7
Untreated
30 µM
80 µM
0h
24h
3)
A) LA-6
B) LA3IK
C) SR-8
D) CR-8
E) RP-7
Untreated
30 µM
80 µM
0h
24h
4)
A) LA-6
B) LA3IK
C) SR-8
D) CR-8
E) RP-7
Untreated
30 µM
80 µM
0h
24h

Figure S5:

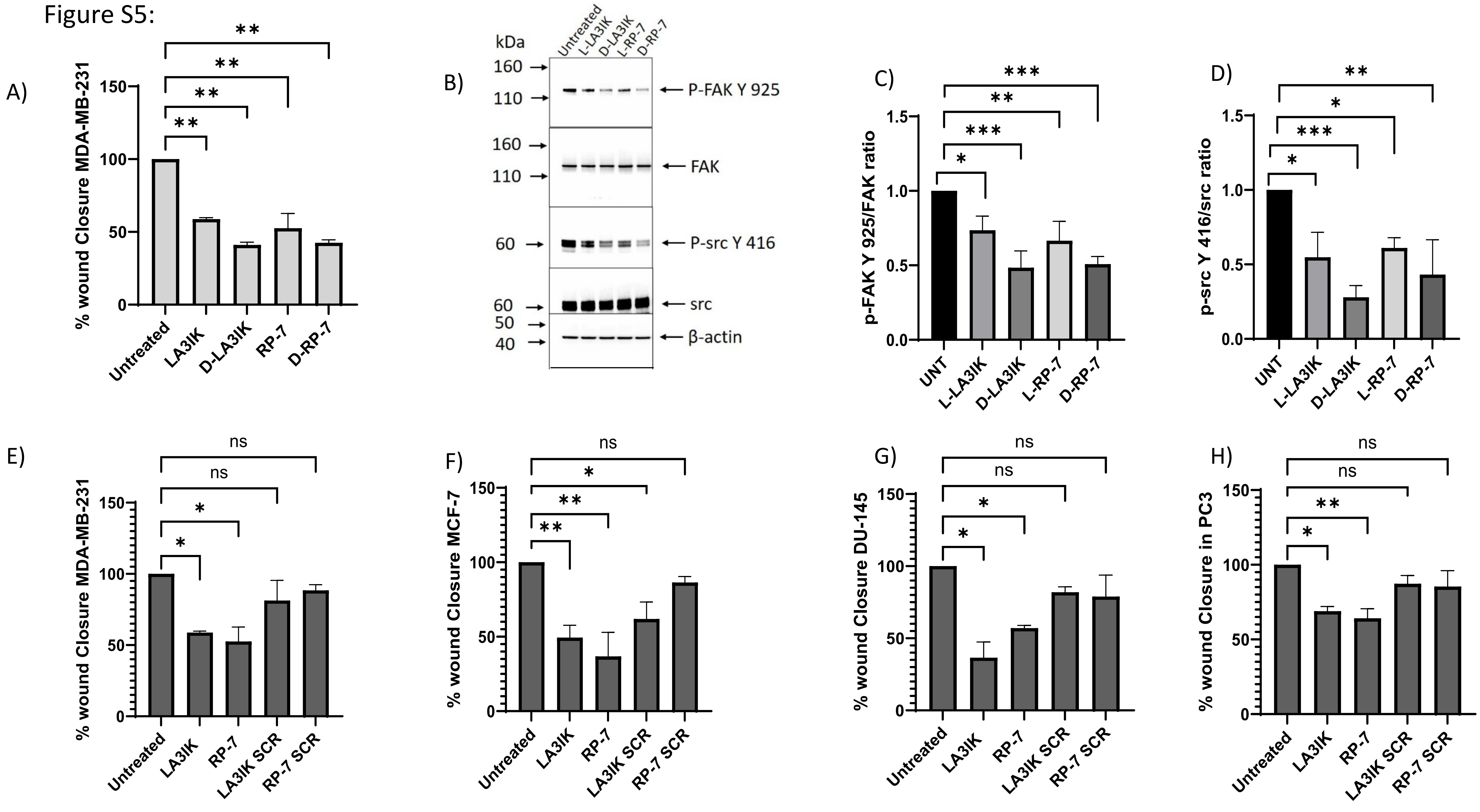
A)
% wound Closure MDA-MB-231
Untreated
LA3IK
D-LA3IK
RP-7
D-RP-7
B)
kDa
Untreated
L-LA3IK
D-LA3IK
L-RP-7
D-RP-7
P-FAK Y 925
FAK
P-src Y 416
src
β-actin
C)
p-FAK Y 925/FAK ratio
UNT
L-LA3IK
D-LA3IK
L-RP-7
D-RP-7
D)
p-src Y 416/src ratio
E)
% wound Closure MDA-MB-231
LA3IK SCR
RP-7 SCR
F)
% wound Closure MCF-7
G)
% wound Closure DU-145
H)
% wound Closure in PC3

Figure S6:

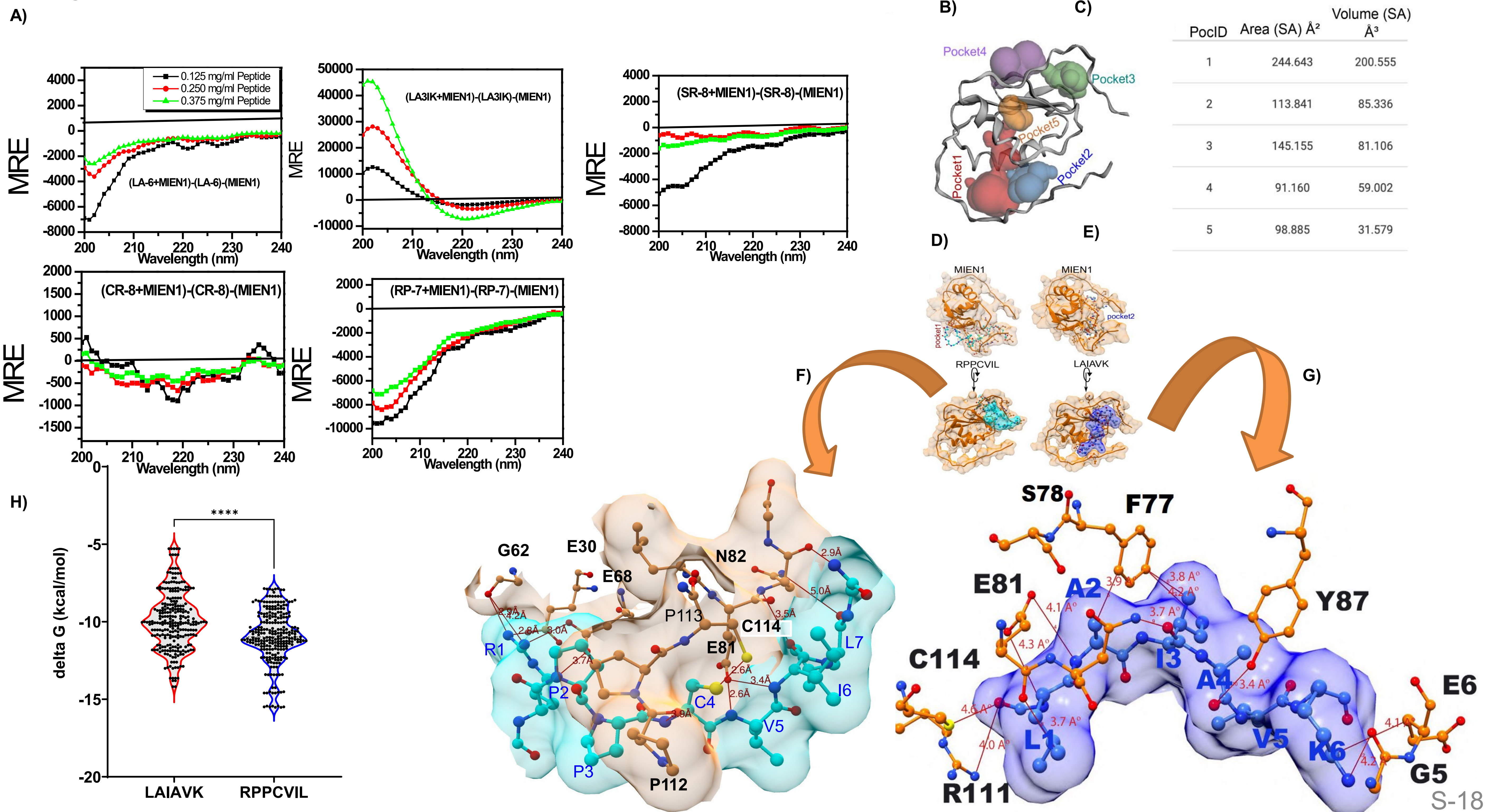

PocID	Area (SA) Å²	Volume (SA) Å³
1	244.643	200.555
2	113.841	85.336
3	145.155	81.106
4	91.160	59.002
5	98.885	31.579

Figure S7(1): A)

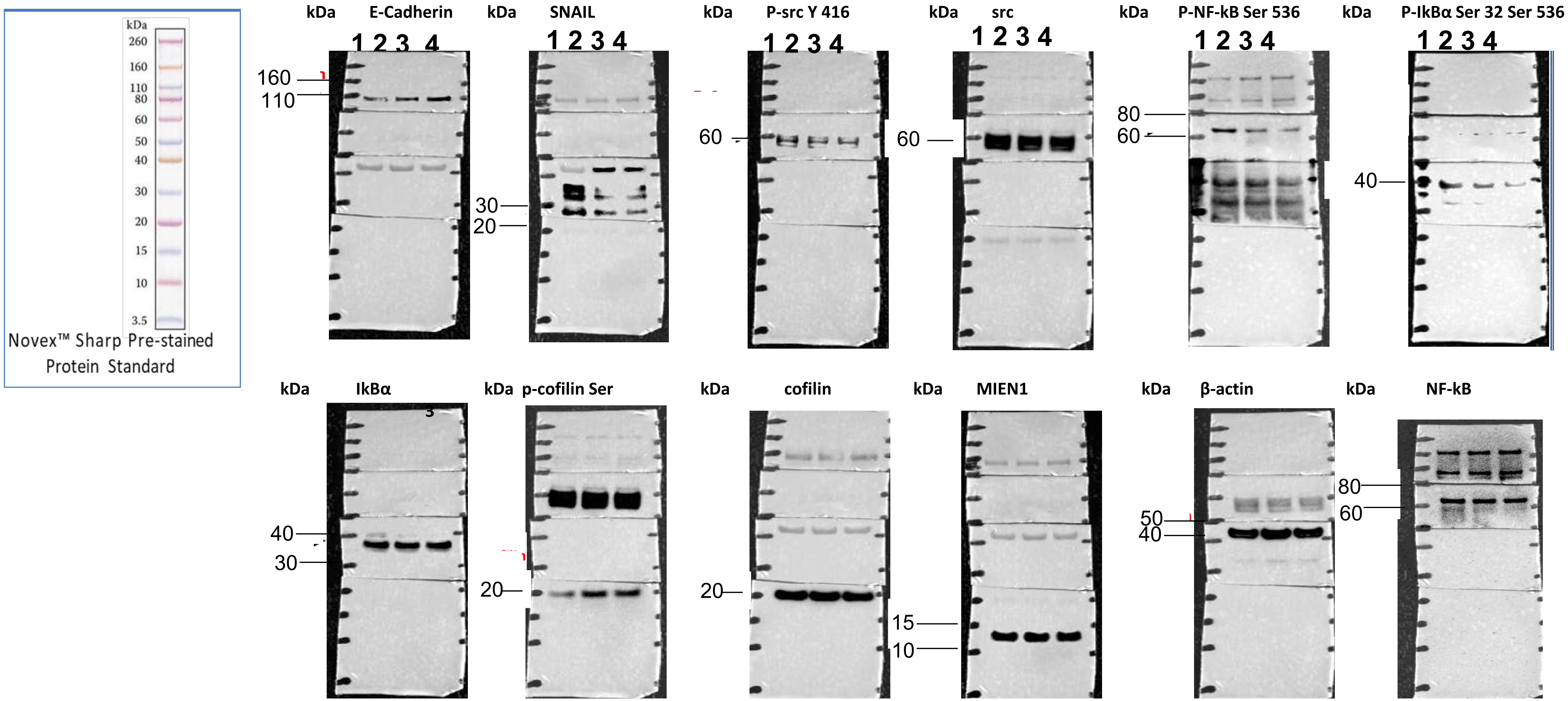

B)

1	Protein Standard Ladder
2	Untreated
3	LA3IK
4	RP-7

Figure S7(2)

A)

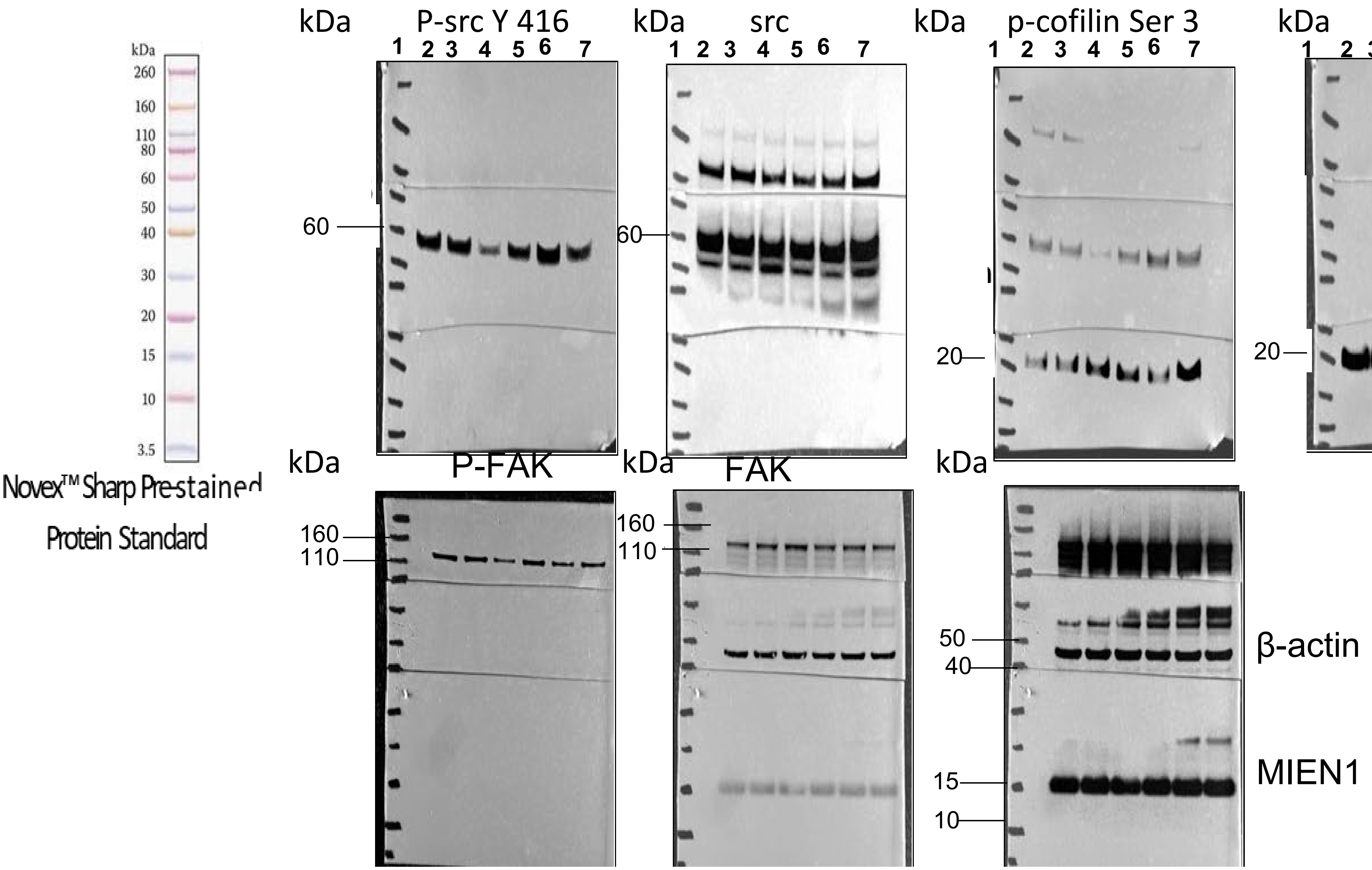

B)

1	**Protein Standard Ladder**
2	**Untreated**
3	**LA-6**
4	**LA3IK**
5	**SR-8**
6	**CR-8**
7	**RP-7**

Figure S8(1):

A)

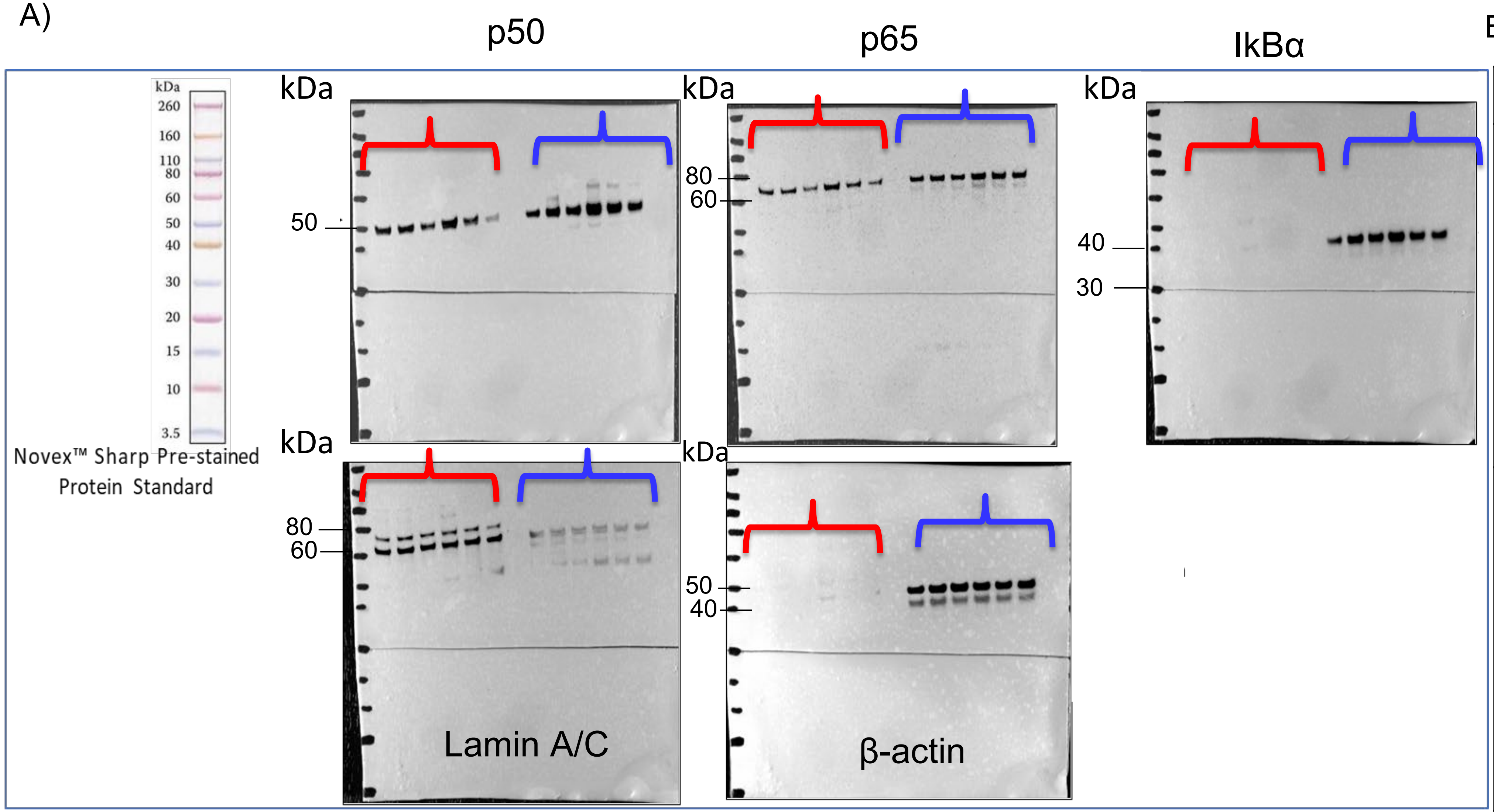

B)

Color Code	Samples (Left to Right)
Nuclear Fraction	EGF Only
	LA-6
	LA3IK
	SR-8
	CR-8
	RP-7
Cytoplasmic Fraction	EGF Only
	LA-6
	LA3IK
	SR-8
	CR-8
	RP-7

Figure S8(2):

A)

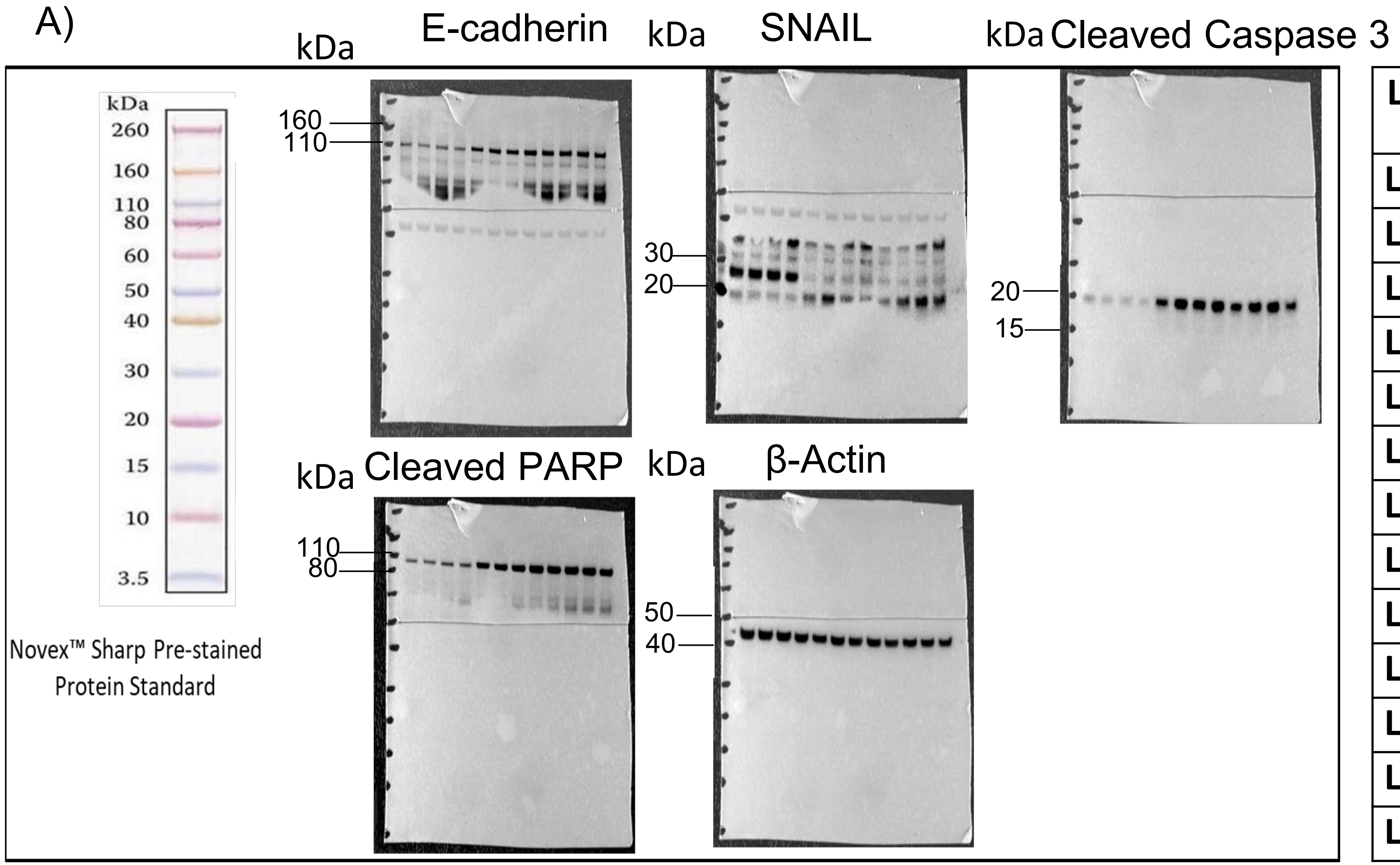

B)

Lane Numbers (Left to Right)	Sample Details
Lane 1	Protein Standard Ladder
Lane 2	Vehicle Tumor 1
Lane3	Vehicle Tumor 2
Lane 4	Vehicle Tumor 3
Lane 5	Vehicle Tumor 4
Lane 6	LA3IK treated Tumor 1
Lane 7	LA3IK treated Tumor 2
Lane 8	LA3IK treated Tumor 3
Lane 9	LA3IK treated Tumor 4
Lane 10	RP-7 treated Tumor 1
Lane 11	RP-7 treated Tumor 2
Lane 12	RP-7 treated Tumor 3
Lane 13	RP-7 treated Tumor 4

References:

1. Tripathi, A. K., Kumari, T., Tandon, A., Sayeed, M., Afshan, T., Kathuria, M. *et al.* (2017) Selective phenylalanine to proline substitution for improved antimicrobial and anticancer activities of peptides designed on phenylalanine heptad repeat Acta Biomater **57**, 170-186 10.1016/j.actbio.2017.05.007
2. Banerjee, P., Eckert, A. O., Schrey, A. K., andPreissner, R. (2018) ProTox-II: a webserver for the prediction of toxicity of chemicals Nucleic Acids Res **46**, W257-W263 10.1093/nar/gky318.
3. Maier, J. A., Martinez, C., Kasavajhala, K., Wickstrom, L., Hauser, K. E., andSimmerling, C. (2015)ff14SB: Improving the Accuracy of Protein Side Chain and Backbone Parameters from ff99SB J Chem Theory Comput **11**, 3696-3713 10.1021/acs.jctc.5b00255.
4. Grosdidier, A., Zoete, V., andMichielin, O. (2011) SwissDock, a protein-small molecule docking web service based on EADock DSS Nucleic Acids Res **39**, W270-277 10.1093/nar/gkr366.
5. Brooks, B. R., Brooks, C. L., 3rd, Mackerell, A. D., Jr., Nilsson, L., Petrella, R. J., Roux, B. *et al.* (2009)CHARMM: the biomolecular simulation program J Comput Chem **30**, 1545-1614 10.1002/jcc.21287.
6. Haberthur, U., andCaflisch, A. (2008) FACTS: Fast analytical continuum treatment of solvation J Comput Chem **29**, 701-715 10.1002/jcc.20832
